# Whole-body CD8^+^ T cell visualization before and during cancer immunotherapy: a phase 1/2 trial

**DOI:** 10.1038/s41591-022-02084-8

**Published:** 2022-12-05

**Authors:** Laura Kist de Ruijter, Pim P. van de Donk, Jahlisa S. Hooiveld-Noeken, Danique Giesen, Sjoerd G. Elias, Marjolijn N. Lub-de Hooge, Sjoukje F. Oosting, Mathilde Jalving, Wim Timens, Adrienne H. Brouwers, Thomas C. Kwee, Jourik A. Gietema, Rudolf S. N. Fehrmann, Bernard M. Fine, Sandra M. Sanabria Bohórquez, Mahesh Yadav, Hartmut Koeppen, Jing Jing, Sebastian Guelman, Mark T. Lin, Michael J. Mamounas, Jeffrey Ryan Eastham, Patrick K. Kimes, Simon P. Williams, Alexander Ungewickell, Derk J. A. de Groot, Elisabeth G. E. de Vries

**Affiliations:** 1 Department of Medical Oncology, University Medical Center Groningen, University of Groningen, Groningen, the Netherlands; 2 Clinical Pharmacy and Pharmacology, University Medical Center Groningen, University of Groningen, Groningen, the Netherlands; 3 Department of Epidemiology, Julius Center for Health Sciences and Primary Care, University Medical Center Utrecht, Utrecht University, Utrecht, the Netherlands; 4 Department of Pathology and Medical Biology, University Medical Center Groningen, University of Groningen, Groningen, the Netherlands; 5 Medical Imaging Center, University Medical Center Groningen, University of Groningen, Groningen, the Netherlands; 6 Genentech Inc., South San Francisco, CA USA

**Keywords:** Cancer imaging, Tumour immunology, Cancer immunotherapy

## Abstract

Immune checkpoint inhibitors (ICIs), by reinvigorating CD8^+^ T cell mediated immunity, have revolutionized cancer therapy. Yet, the systemic CD8^+^ T cell distribution, a potential biomarker of ICI response, remains poorly characterized. We assessed safety, imaging dose and timing, pharmacokinetics and immunogenicity of zirconium-89-labeled, CD8-specific, one-armed antibody positron emission tomography tracer ^89^ZED88082A in patients with solid tumors before and ~30 days after starting ICI therapy (NCT04029181). No tracer-related side effects occurred. Positron emission tomography imaging with 10 mg antibody revealed ^89^ZED88082A uptake in normal lymphoid tissues, and tumor lesions across the body varying within and between patients two days after tracer injection (*n* = 38, median patient maximum standard uptake value (SUV_max_) 5.2, IQI 4.0–7.4). Higher SUV_max_ was associated with mismatch repair deficiency and longer overall survival. Uptake was higher in lesions with stromal/inflamed than desert immunophenotype. Tissue radioactivity was localized to areas with immunohistochemically confirmed CD8 expression. Re-imaging patients on treatment showed no change in average (geometric mean) tumor tracer uptake compared to baseline, but individual lesions showed diverse changes independent of tumor response. The imaging data suggest enormous heterogeneity in CD8^+^ T cell distribution and pharmacodynamics within and between patients. In conclusion, ^89^ZED88082A can characterize the complex dynamics of CD8^+^ T cells in the context of ICIs, and may inform immunotherapeutic treatments.

## Abstract (web summary)

A CD8-specific, one-armed antibody positron emission tomography tracer enables the visualization of the immune response in patients with solid tumors before and after starting immunotherapy.

## Main

T cell-enhancing immune checkpoint inhibitors (ICIs) have gained their place in cancer treatment with impressive, durable antitumur efficacy in a remarkable variety of tumor types^1–3^. However, response rates vary, and only a subset of patients benefits. A combination with another ICI or other medicines can improve response rates but can also increase the risk of adverse events (AEs)^1^. This highlights the clinical need for tools to optimize treatment strategies for individual patients. Several biomarkers have been identified to select patients for ICI^3^. These include programmed death-ligand 1 (PD-L1) expression, tumor mutational burden, deficiency of mismatch repair (dMMR) proteins and a T cell-inflamed gene expression profile^4–6^. However, no single biomarker or combination of biomarkers accurately predicts response to ICI.

CD8^+^ T cells play an essential role in tumor cell destruction by the immune system. Their presence in the tumor is associated with responses to ICIs across several tumor types^6–10^. An ICI treatment-emergent increase in CD8^+^ T cell density in tumor biopsy samples has also been associated with tumor response. Most data are available for patients with advanced melanoma with biopsy samples obtained at different time points following the start of ICIs. For example, increased CD8^+^ cell density in 25 paired tumor biopsy samples collected after 20–120 days pembrolizumab treatment was associated with response^11^. Others reported a CD8^+^ T cell expansion in 13 biopsy samples two weeks after anti-programmed cell death (PD-1) antibody therapy initiation, but this was not the case in a study analysing ten mostly late on-treatment biopsy samples after 0.7–26 months^9,10^. Sampling bias may influence these differences and considerable heterogeneity can exist within or between different lesions within one patient^12,13^.

Due to these inherent limitations for invasive tumor biopsies, remarkably little is known about the systemic kinetics and heterogeneity of CD8^+^ T cell distribution among tumor types and individual tumor lesions in patients. To address this issue, we developed the zirconium-89-labeled one-armed antibody ^89^ZED88082A targeting CD8a, as antibodies or antibody fragments labeled with zirconium-89 (^89^Zr) allow noninvasive whole-body visualization of a target with positron emission tomography (PET)^14–16^. First, ^89^ZED88082A uptake with PET was shown in human CD8-expressing tumors xenografted in mice^17^. We then performed ^89^ZED88082A PET scanning in patients with solid tumors before and ~30 days after starting ICI treatment with PD-L1 antibody, or PD-1 antibody with or without CTLA-4 antibody. The primary objectives of the study were to characterize the safety, imaging dose and time points, pharmacokinetics and immunogenicity of ^89^ZED88082A in patients with solid tumors. Secondary objectives included the potential to image whole-body CD8^+^ T cells, correlations of CD8 PET imaging data with tumor-based assessments and correlations with clinical outcomes and AE to ICI treatment.

## Results

### Trial population and safety

Between February 2019 and November 2020, 39 patients were enrolled (NCT04029181). One patient with tracer extravasation was excluded from PET analyses (Table 1). Twenty-two of the 29 consecutive patients included for repeated imaging did undergo this, with a median of 30 days following initiation of ICI treatment (IQI 28–36 days). Seven were not scanned during ICI therapy, because of withdrawal before (*n* = 1) and during (*n* = 4) treatment due to disease progression, patient anxiety (*n* = 1) and COVID-19 restrictions (*n* = 1).

**Table 1:** Characteristics at study entry of all evaluable patients

Characteristics	Sample, total *n* = 38
**Median age**, years (range)	62 (32–80)
**Sex**, *n* (%)	
Female	20 (53)
Male	18 (47)
**Tumor types**, *n* (%)	
dMMR (colorectal 5 (13%), UCC 2 (5%), duodenal 1 (3%), pancreatic 1 (3%))	9 (24)
Cervical carcinoma	5 (13)
Cutaneous SCC	4 (11)
TNBC	3 (8)
Cholangiocarcinoma	3 (8)
Melanoma	3 (8)
Anorectal SCC	2 (5)
Vulvar SCC	2 (5)
NEC (cervical, gastric-esophageal)	2 (5)
Esophageal SCC	1 (3)
NSCLC	1 (3)
Hepatocellular carcinoma	1 (3)
Ovarian clear cell carcinoma	1 (3)
SCC of unknown primary	1 (3)
**Tumor stage at study entry**, *n* (%)	
Loco-regional irresectable	3 (8)
Metastatic	35 (92)
**ECOG performance status**, *n* (%)	
0	19 (50)
1	19 (50)
**Previous lines of systemic treatment in neo-adjuvant or adjuvant setting**, *n* (%)	
0	32 (84)
1	4 (11)
≥2	2 (5)
**Previous lines of systemic treatment in the locally advanced or metastatic setting***, n* (%)	
0	29 (76)
1	4 (11)
≥2	5 (13)

ECOG, Eastern Cooperative Oncology Group. NEC, neuroendocrine carcinoma. NSCLC, non-small-cell lung cancer. SCC, squamous cell carcinoma. TNBC, triple-negative breast cancer. UCC, urothelial cell carcinoma.

No ^89^ZED88082A-related side effects occurred. AEs due to ICI were consistent with reports from previous studies (Extended Data Table 1).

**Fig. 1:**
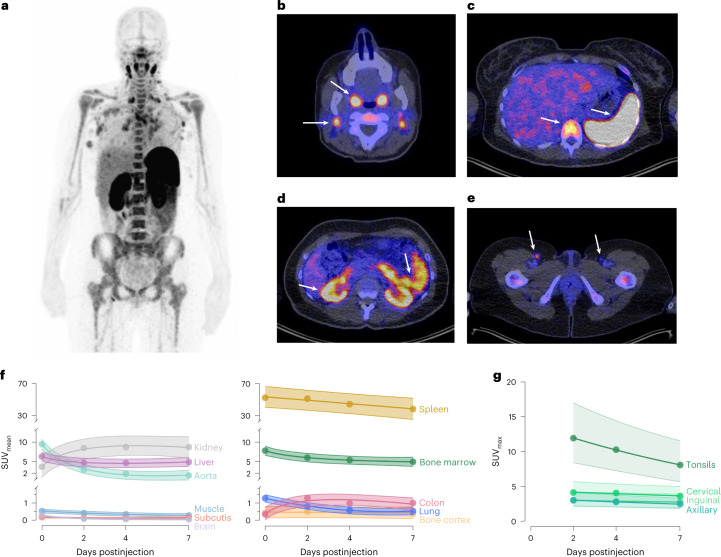
Normal tissue biodistribution of ^89^ZED88082A. **a**, Representative ^89^ZED88082A PET scan maximum intensity projection day 2. A whole-body visualization is available as Supplementary Video. **b–e**, Axial views of the same scan fused with low-dose CT. Arrows indicate uptake in Waldeyer’s ring, cervical lymph nodes (**b**), spleen, bone marrow (**c**), renal cortex, small intestine (**d**) and inguinal lymph nodes (**e**). **f**,**g** Pretreatment uptake with 95% confidence bands across tissues adjusted for protein dose, projected at 10 mg dose (*n* = 9), days 0 (1 h), 2, 4 and 7 (±1 day), with mean SUV_mean_ (**f**) and mean SUV_max_ (**g**) for lymph nodes and tonsils, not visible on day 0.

**Extended Data Fig. 1:**
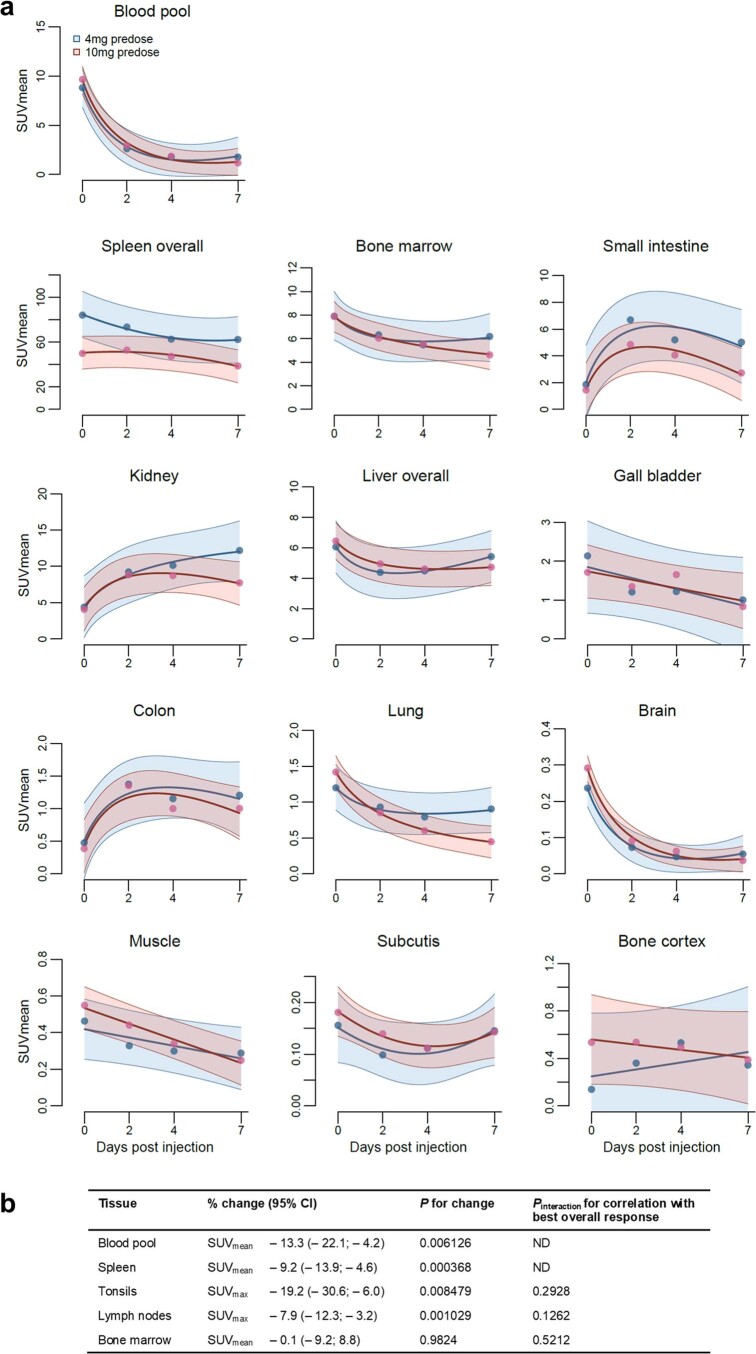
Biodistribution of ^89^ZED88082A in normal tissues. **a**, Biodistribution per protein dose cohort pretreatment. Graphs show the average SUV_mean_ with 95% confidence bands of ^89^ZED88082A in the blood pool and normal tissues at days 0 (1 h), 2, 4, and 7. Colours reflect the dose cohorts with 4 mg in blue (*n* = 3) and 10 mg in red (*n* = 6). Note the different scales of the y-axis. **b**, Table showing average changes in tracer uptake values between pre- and ontreatment PET scans, projected at 4 weeks. P_interaction_ is shown for the correlation between change and best overall response (PD vs. SD/PR/CR). ND = not determined.

**Extended Data Fig. 2:**
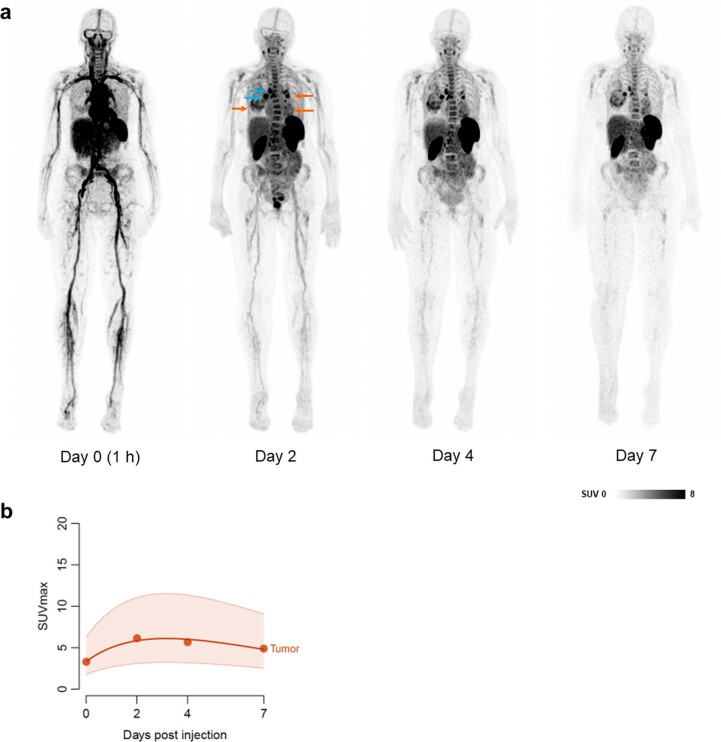
^89^ZED88082A biodistribution in time. **a**, Whole-body maximum intensity projection (MIP) images of a patient with cervical cancer show biodistribution of ^89^ZED88082A over time at 10 mg tracer dose. Orange arrows indicated lung metastases; blue arrows indicate uptake in hilar lymph nodes. **b**, ^89^ZED88082A tumor uptake pretreatment projected at 10 mg tracer dose as geometric mean SUV_max_ (line and dots) with 95% confidence bands for all nine patients in part A (lesions *n* = 70).

**Extended Data Fig. 3:**
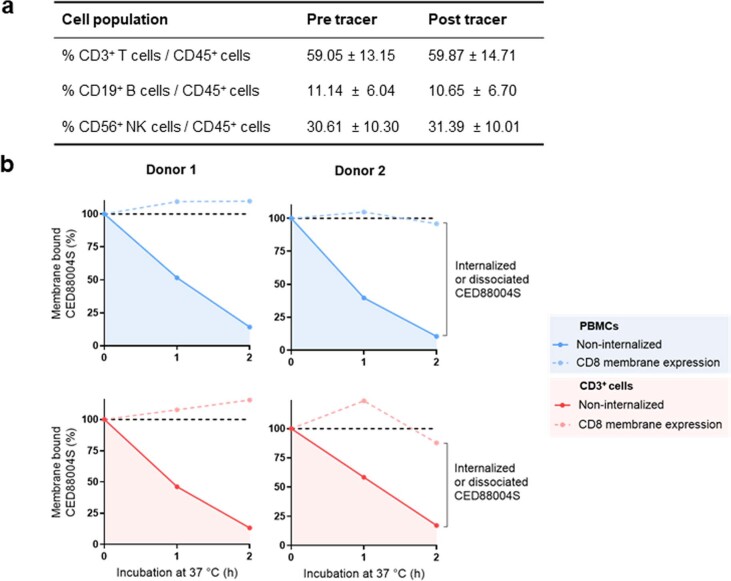
^89^ZED88082A/CED88004S effects in PBMCs. **a**, Table shows no difference in mean ± SD for T cell, B cell, and NK cell counts in blood samples from patients before (day 0) and post-tracer injection (day 2), at baseline before the start of ICI. **b**, CED88004S internalization experiments in PBMCs of healthy donors. CED88004S binding to CD8 and subsequent internalization was determined by flow cytometry in unstimulated PBMCs from healthy blood donor buffy coat. Membrane-bound CED88004S was detected using an anti-human allophycocyanin-IgG F(ab′)2 fragment. CD8 membrane levels before incubation (T = 0 h) were set at 100%. Blue: Internalization in total PBMC population, Red: Internalization in CD3 positive cells. CD8-bound CED88004S on the cell surface decreased during incubation at 37 °C (solid line), while membrane levels remained stable (dashed line).

In part A, two anti-CD8 tracer protein doses (^89^ZED88082A + unlabeled, desferrioxamine (DFO)-conjugated one-armed antibody CED88004S) were evaluated: 4 mg (*n* = 3) or 10 mg (*n* = 6) with serial PET scans 0 (1 h), 2, 4 and 7 (±1) days after administration, followed by a biopsy of a tumor lesion. The 10 mg dose allowed for sufficient blood pool tracer availability (average day 2 mean standard uptake value (SUV_mean_) 2.9 (±1.0), day 4 SUV_mean_ 1.9 (±0.3)). Compared to 4 mg, the 10 mg dose showed less and stable splenic uptake, indicating abatement of splenic tracer sink effect (Extended Data Fig. 1a). The 10 mg protein dose visualized tumor lesions and lymphoid tissues (Fig. 1 and Supplementary Video), with highest uptake on days 2 and 4 (Extended Data Fig. 2). In vitro, human peripheral blood mononuclear cells did not internalize the tracer (Extended Data Fig. 3), consistent with PET imaging data showing no further increase in tissue signal between days 2–7. Therefore, in part B, the 10 mg protein dose with PET scanning on day 2 was considered optimal.

### Uptake in tumor lesions at baseline

**Fig. 2:**
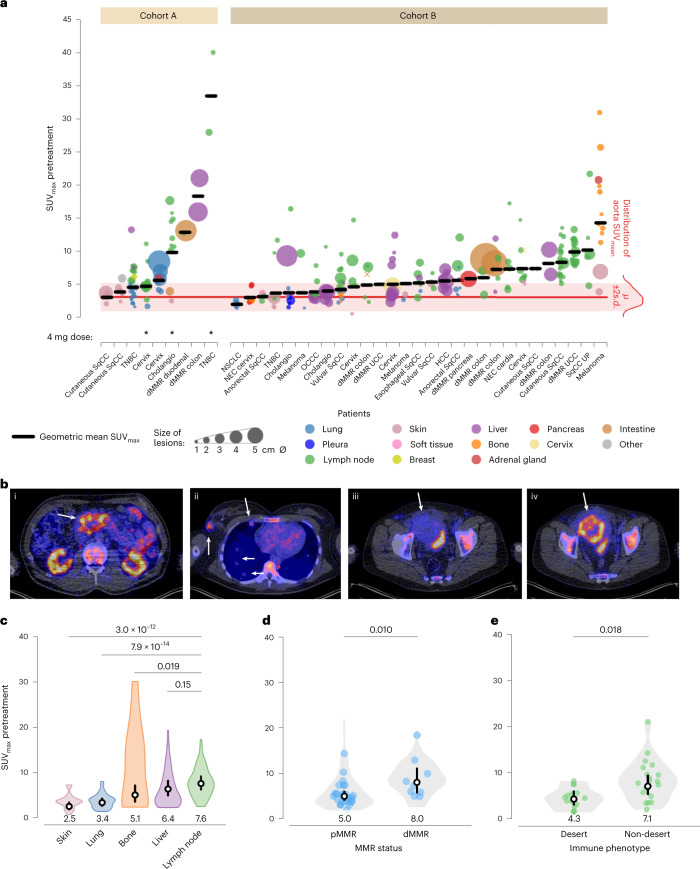
^89^ZED88082A uptake in nonirradiated tumor lesions. **a**, Pretreatment uptake in 266 lesions day 2 after tracer injection, ordered by increasing geometric mean SUV_max_ per patient, visualizing lesion size and site, and aorta background uptake. ∅, diameter. *μ*, mean. **b**, Axial views PET/CT scans, arrows indicate lesions. (i) High, heterogeneous uptake in dMMR duodenal tumor. (ii) Uptake in a triple-negative right breast cancer lesion, moderate uptake in pleural and no to minor uptake in lung lesions. (iii) Minor uptake in perivesical dMMR urothelial cell cancer lesion pretreatment increased with rim pattern during treatment (iv). **c**, Violin plot SUV_max_ in lesions (*n* = 212) per site (lymph nodes *n* = 99, liver *n* = 35, bone *n* = 17, lung *n* = 42, skin *n* = 19). **d**, Violin plot of SUV_max_ in patients with pMMR (*n* = 25) and dMMR tumors (*n* = 9). **e**, Violin plot of SUV_max_ in lesions with desert (*n* = 15) and nondesert (*n* = 19) immune phenotype before and during treatment in 24 patients. **c–e**, Violin plots with bottom and top 1% of SUV_max_ values truncated (**c** and **d**, not for **e**); colored dots are the geometric means per patient (**d**) or lesion (**e**); black vertical lines are geometric mean SUV_max_ 95% CI; white dots within black lines and values below the violin plot the actual geometric means. Two-sided nominal *P* values were derived from linear mixed models taking clustering within patients (and, if applicable, lesions) into account, using a Wald test under restricted maximum likelihood for three of higher-level factors (**c**) or a likelihood ratio test under maximum likelihood for two-level factors (**d**,**e**). SqCC, squamous cell carcinoma; OCCC, ovarian clear cell carcinoma; HCC, hepatocellular carcinoma; UP, unknown primary.

**Extended Data Fig. 4:**
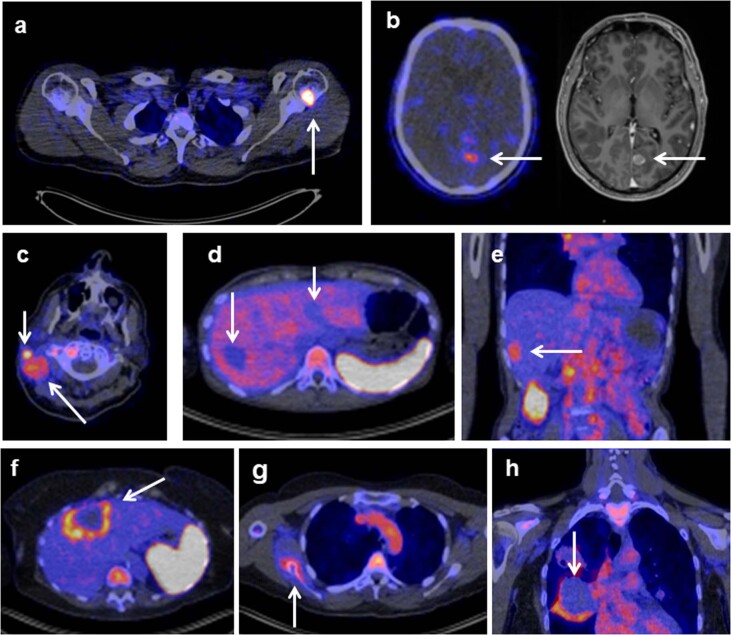
PET image examples of uptake in tumor lesions. **a**, Bone metastasis with high tracer uptake (SUV_max_ 18.9) in a patient with melanoma. **b**, Uptake in a brain metastasis (SUV_max_ 1.6) of a patient with melanoma with corresponding MRI, whereas healthy brain showed low uptake with SUV_mean_ 0.1. **c**, High uptake in multiple cervical lymph node metastases in a patient with cutaneous squamous cell carcinoma. **d**, Multiple liver metastases in a patient with ovarian clear cell carcinoma without ^89^ZED88082A uptake. **e**, Uptake in a liver metastasis in a patient with squamous cell oesophageal cancer. **f-h**, Several metastases with high rim uptake: **f**, Liver metastases in a patient with dMMR colorectal cancer. **g**, Bone lesion in a patient with squamous cell vulvar cancer. **h**, Lung metastasis in a patient with cervical cancer.

Baseline ^89^ZED88082A uptake in all nonirradiated lesions (*n* = 266 in 38 patients) showed an overall geometric mean SUV_max_ of 5.6 (geometric coefficient of variation 0.72) on day 2. Lesions were detected in all major organs. Median geometric mean SUV_max_ per patient was 5.2 (IQI 4.0–7.4). Heterogeneity in tumor uptake was observed between and within patients (intraclass correlation coefficient 0.46; Fig. 2a,b(ii) and Extended Data Fig. 4). In 10 patients, 4 with dMMR tumors, 16 lesions (6 dMMR) showed a pronounced tumor-rim uptake (Fig. 2b and Extended Data Fig. 4f–h). Among the 13 evaluable lesions out of these 16, only 3 had computed tomography (CT) evidence of central necrosis.

**Fig. 3:**
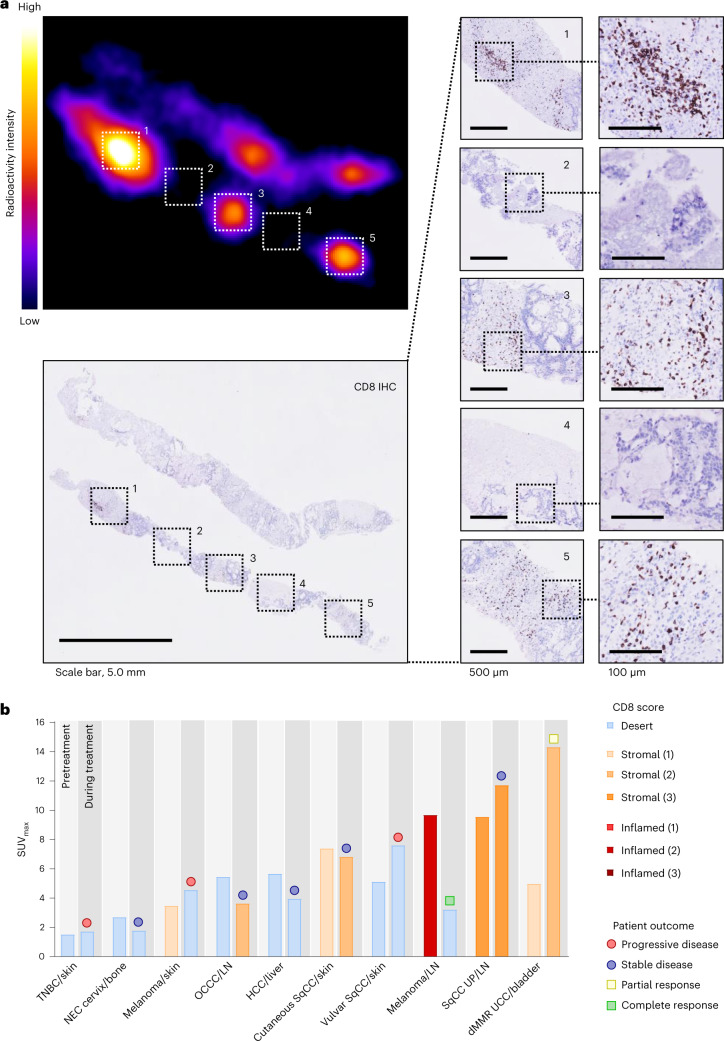
^89^ZED88082A in tumor tissues related to CD8 by IHC. **a**, Autoradiography image of ^89^ZED88082A uptake in a dMMR colorectal cancer liver metastasis and accompanying CD8 IHC staining. Areas 1, 3 and 5 with moderate to high CD8 expression; 2 and 4 without CD8 expression. The representative image is shown with evident correlation between IHC CD8 expression and autoradiography signal (*n* = 16). **b**, Overview of SUV_max_ and CD8 IHC expression pattern (density score) in lesions with corresponding paired biopsy samples before and during treatment in ten patients. On the *x* axis, primary tumor type and location of biopsy are shown. The symbol above the bar indicates the radiographic response of the lesion at six weeks. LN, lymph node. Source data

**Extended Data Fig. 5:**
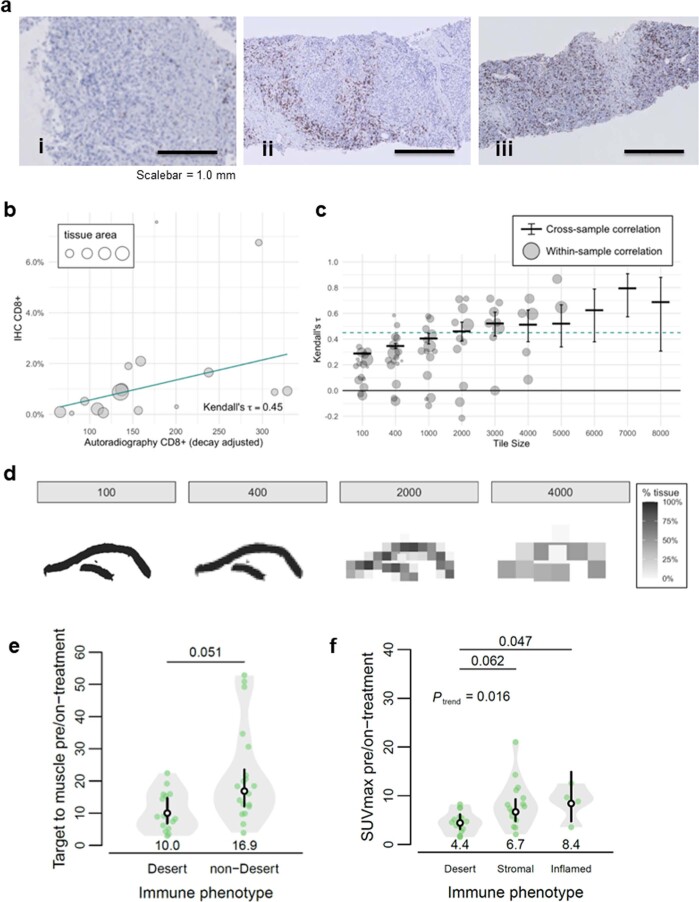
Tumor tissue IHC analyses and correlation with tracer signal. **a**, Representative examples of IHC CD8 expression phenotypes (*n* = 34): i, Liver biopsy of a cholangiocarcinoma metastasis with a desert phenotype. ii, A biopsy of a perivesical tumor mass of dMMR urothelial cell cancer with stromal CD8 expression phenotype [density 2]. iii, A liver biopsy of dMMR colon carcinoma shows an inflamed phenotype [density 3]. **b**, Correlation of mean CD8 staining pixel positivity and autoradiography signal across 16 samples with weighted quantile regression fit. Point sizes and regression weights are proportional to the size of each sample biopsy. **c**, Using tile-based analysis, the correlation across and within samples of mean CD8 staining pixel positivity and autoradiography signal at subsample level. Cross-sample correlations and corresponding 95% confidence intervals are displayed with horizontal and vertical lines at each tile size. Within-sample correlations are presented at each tile size for each sample as circles. At each tile size, only samples with ≥ 6 tiles are shown. Tiles containing less than < 25% tissue were excluded. Only cross-sample correlations are shown at tiles sizes higher than 5000px2 as no single sample had >5 tiles. **d**, Tiles of varying sizes are shown for a single representative clear cell ovarian cancer sample. **e**, Violin plot of tumor SUV_max_-to-muscle SUV_mean_ ratio with desert (*n* = 15) and non-desert (*n* = 19) immune phenotype before and during treatment in 24 patients. **f**, Violin plot of SUV_max_ in lesions with desert (*n* = 15), stromal (*n* = 15) and inflamed (*n* = 4) immune phenotype before and during treatment in 24 patients. **e-f**, Coloured dots show individual lesions; black vertical lines show 95% CI of the geometric mean; white dots within black lines and values below the violin plot the actual geometric means. Two-sided nominal *P*-values were derived from linear mixed models taking clustering within patients into account, either using a likelihood ratio test under maximum likelihood (e; P_trend_ in f) or using a Wald test under restricted maximum likelihood (*P*-values for factor levels in f).

^89^ZED88082A uptake was related to the lesion’s organ location and highest in malignant lymph nodes (Fig. 2c). Malignant lymph nodes also exhibited 62% higher SUV_max_ than normal lymph nodes (95% confidence interval (CI) 45–80%, *P* ≤0.001). We took two approaches to verify whether potential differences in CD8 tracer uptake did reflect CD8-related tumor characteristics. First, we showed that ^89^ZED88082A tumor uptake was higher in the 9 patients with dMMR than the 25 with mismatch repair proficient (pMMR) tumors (Fig. 2d). Second, we studied with CD8 immunohistochemistry (IHC) the tumors of 24 patients with 22 pre- and 12 on-treatment samples. This showed four inflamed, 15 stromal and 15 desert phenotypes (Extended Data Fig. 5a and Supplementary Fig. 1). The SUV_max_ was higher in inflamed or stromal phenotype lesions than desert phenotype lesions before and during treatment (Fig. 2e and Extended Data Fig. 5f). Lesions with a CD8 desert phenotype had a geometric mean SUV_max_ of 4.3 (95% CI 3.1–6.0), while lesions with a stromal or inflamed phenotype had a geometric mean SUV_max_ of 7.1 (95% CI 5.4–9.4) (*P* = 0.018); when presented as a to the physiological muscle background uptake, this difference was not significant (Extended Data Fig. 5e). Localized CD8^+^ T cell density by IHC correlated with the autoradiography signal magnitude in tumor tissues (*τ* = 0.45, *P* = 0.015) (Fig. 3a and Extended Data Fig. 5b–d).

**Fig. 4:**
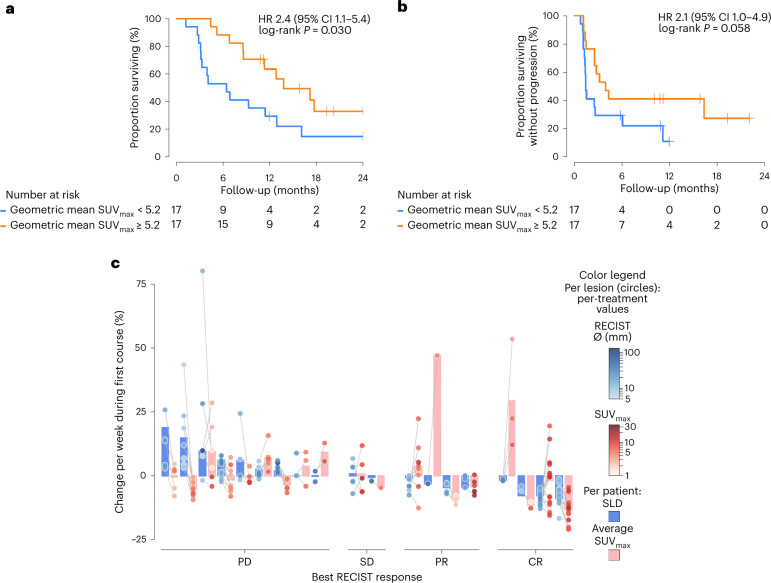
^89^ZED88082A uptake related to tumor response. **a**,**b,** PFS (**a**) and OS (**b**), according to baseline geometric mean SUV_max_ below and above median, and with two-sided nominal *P* values derived from a log-rank test. **c**, Changes during repeated imaging in tumor uptake and anatomic size, expressed as estimated changes per week treatment to account for variation between patients in the timing of the PET scan/CT response evaluation. Patients (*n* = 19) are represented by two bars (blue and pink) and grouped per best overall treatment response. Blue bars, change in sum target lesions according to RECIST between pretreatment and first response evaluation. Pink bars, average SUV_max_ change. Dots are individual lesions (*n* = 111). Individual lesion datapoints for size (blue) and uptake (red) are connected by gray lines. Blue dots, lesion blueness, RECIST diameter pretreatment. Dot location, change in size versus baseline. Red dots, lesion redness, SUV_max_ pretreatment. Dot location, SUV_max_ change.

**Extended Data Fig. 6:**
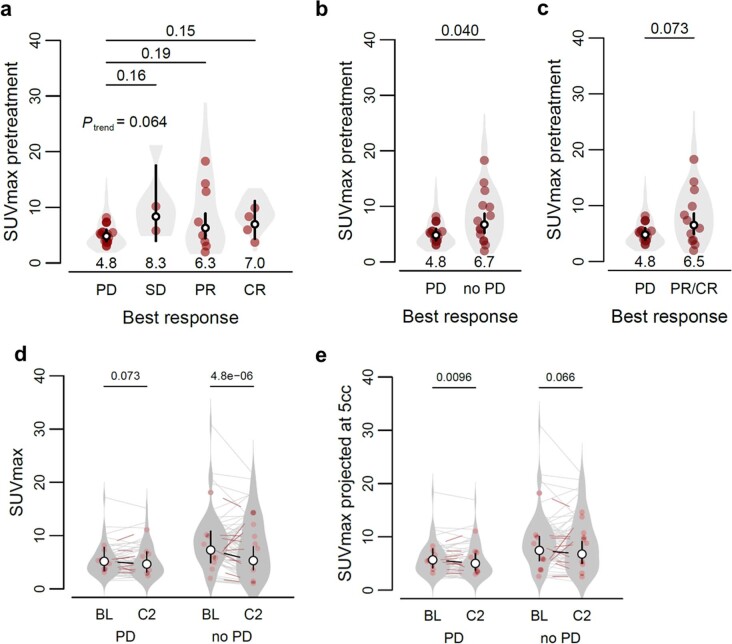
^89^ZED88082A tumor uptake in relation to response. **a**, Relationship between pretreatment ^89^ZED88082A uptake and best overall response; red dots show geometric mean SUV_max_ per patient. Violin plot areas show actual distribution of SUV_max_ at the metastasis level per category, white dots and values below the violin plot show geometric means, black vertical lines show 95% CIs of geometric mean SUV_max_ (PD: 149 lesions in 19 patients; SD: 6 lesions in two patients, PR: 41 lesions in eight patients; CR: 36 lesions in four patients). **b**, Relationship between pretreatment ^89^ZED88082A uptake in patients with progressive disease and those that did not progress. **c**, Relationship between pretreatment ^89^ZED88082A uptake in patients with progressive disease and those with PR or CR, excluding SD. **a-c**, Two-sided nominal *P*-values were derived from linear mixed models taking clustering within patients into account, using a Wald test under restricted maximum likelihood for three or higher-level factors or a likelihood ratio test under maximum likelihood for two-level factors and for the trend test. **d-e**, Changes in tumor lesion SUV_max_ between the pre- and on-treatment PET scans. Patients are grouped per best overall response (PD, or no-PD). Violin plots show actual distribution of individual lesions. Baseline (BL) to response scan 1 (RS1, on-treatment) trajectories of individual lesions are shown with grey lines, projected at 30 days, when PET scan was regularly performed; red lines and dots (geometric means) present per-patient aggregated data; white dots are the overall geometric means with black 95% CI bars. Two-sided nominal *P*-values were derived from linear mixed models taking clustering within patients and lesions into account, using a Wald test under restricted maximum likelihood. **d**, Compared to pre-treatment, patients with SD, PR, or CR show a lowered uptake on the on-treatment PET scan than those with PD (*P*_interaction_ = 0.018). **e**, Same change in SUV_max_, projected at 5 cm^3^ tumour volume to adjust for volume changes (*P*_interaction_ = 0.71).

As of 13 October 2021, median patient follow-up was 5.6 months; 35 of 38 patients were evaluable for best overall response, 4 patients experienced a complete response (CR), 8 a partial response (PR), 4 stable disease (SD) and 19 progressive disease (PD). Baseline tracer tumor uptake showed a positive trend with best overall response evaluation criteria in solid tumours (RECIST) response (*P*_trend_ = 0.064, Extended Data Fig. 6a), and uptake was 40% (95% CI 0–94%) higher in patients with SD/PR/CR as best overall response during ICI (*P* = 0.040; Extended Data Fig. 6b,c). Patients with an above-median baseline ^89^ZED88082A-uptake geometric mean SUV_max_ (that is, >5.2) showed a trend towards superior progression-free survival (PFS) (median 1.5, 95% CI 1.3 to not reached; versus 3.9, 95% CI 2.6 to not reached, *P* = 0.058) and had superior overall survival (OS) to patients with an uptake below the median (median 6.5, 95% CI 3.3 to not reached, versus 13.8, 95% CI 11.3 to not reached, *P* = 0.030) (Fig. 4). Analyzed continuously, baseline ^89^ZED88082A-uptake geometric mean SUV_max_ (per standard deviation decrease) showed for PFS a hazard ratio (HR) of 1.60 (95% CI 1.03–2.78; *P* = 0.034) and for OS that of 1.59 (95% CI 1.04–2.72; *P* = 0.031).

### Uptake in tumor lesions during treatment

During treatment, the average ^89^ZED88082A uptake in nonirradiated lesions in all patients (lesion *n* = 111) was lower compared to baseline (−4.6% change in geometric mean SUV_max_ per week of treatment, 95% CI −6.5% to −2.6%), a change that depended on best overall response with a greater decrease in patients with SD, PR or CR (*P*_interaction_ = 0.018) (Extended Data Fig. 6c). Of the eight patients who showed PR or CR on treatment, five already met criteria for PR at the time of the PET scan at 30 days. When taking into account tumor volume change and resulting tracer uptake underestimation due to partial volume effects in responding lesions, the estimated average tracer uptake change was −2.7% (95% CI −4.4% to −1.1%) per week treatment, which no longer depended on best overall response (*P*_interaction_ = 0.71) (Extended Data Fig. 6d). No patient in the repeat imaging cohort experienced pseudoprogression.

Within patients, lesions demonstrated diverse changes in ^89^ZED88082A uptake, with some decreasing and others increasing compared to baseline. Moreover, responding lesions displayed a variety of dynamics in ^89^ZED88082A-uptake change between the two PET series (Fig. 4c).

For ten patients, paired tumor tissues of the same lesion with corresponding tumor volumes of interest (VOIs) on PET were available (Supplementary Information Fig. S1). Five of them reflected concordant treatment-emergent changes by IHC and imaging (Fig. 3b). In one patient, a lymph node metastasis with a SUV_max_ of 8.28 and stromal CD8 T cell infiltration at baseline showed only normal lymph node tissue in the second biopsy sample, with SUV_max_ of 5.63 on the on-treatment PET.

### Normal tissue biodistribution and pharmacokinetics

**Extended Data Fig. 7:**
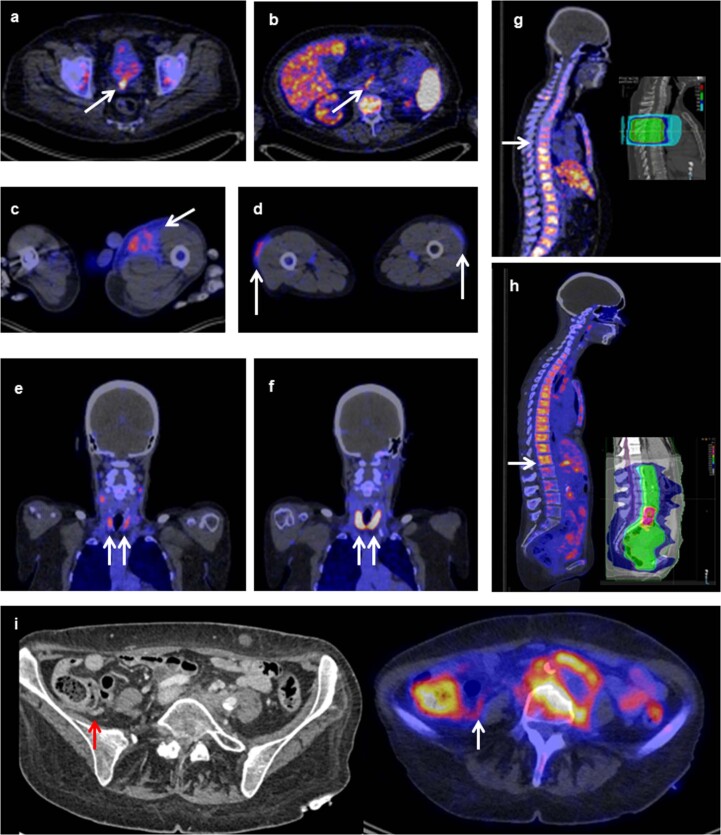
PET image examples of uptake in non-malignant sites. Axial (**a-d, i**), coronal (**e, f**), and sagittal (**g, h**) views of ^89^ZED88082A PET scans with low dose CT. **a**, High uptake in urinary bladder diverticulum with urolithiasis and accompanying inflammation. **b**, Uptake in an aortic atherosclerotic plaque, also detectable on non-attenuated corrected images (data not shown). **c**, Uptake post-surgery in a patient after inguinal lymph node dissection with a seroma. **d,** Bilateral uptake in subcutaneous inflammation reaction on heparin injections. **e**, Hashimoto’s thyroiditis with high uptake before treatment and **f**, Increased uptake during treatment experiencing a flare-up. **g-h**, Two patients who received radiotherapy to the spine; arrows indicate the border of the radiation field; the insert shows the radiation field. The irradiated bone marrow in the spine, shows less uptake than non-irradiated bone marrow. **g**, Patient who received 5×4 Gy to the spine for painful bone metastases 3 months before ^89^ZED88082A PET scan. **h**, Patient who received 25×1.8 Gy on para-aortal lymph nodes alongside the spine 12 months before the ^89^ZED88082A PET scan. **i**, Uptake of tracer in normal appendix on PET (right), with corresponding diagnostic CT (left).

^89^ZED88082A showed a specific uptake per organ (Fig. 1). The highest ^89^ZED88082A uptake occurred in the spleen and was apparent within an hour of injection. From day 2 onwards, there was a clear ^89^ZED88082A uptake in normal lymphoid tissues, including the bone marrow, Waldeyer’s ring, lymph nodes, the small intestine (Extended Data Fig. 1) and the appendix (Extended Data Fig. 7i). Sites with previous lymph node dissection lacked uptake. Furthermore, tracer uptake was present in the renal cortex and liver. Partial volume effects and spillover signal precluded the quantification of small tumor lesions contained within the renal cortex and the spleen. Tracer uptake was also observed at sites of inflammation (Extended Data Fig. 7). In two patients, ^89^ZED88082A uptake was lower in vertebrae irradiated <12 months earlier than in nonirradiated vertebrae (Extended Data Fig. 7g,h). During treatment, the average tracer SUV_mean_ in blood pool at four weeks was 13.3% lower compared to pretreatment. Equally, uptake in spleen and lymphoid tissues was limitedly decreased, the latter not being correlated to best overall response (Extended Data Fig. 1b).

Several patients developed immune-related AEs (irAE) after ICI initiation (Extended Data Table 1). One patient with Hashimoto’s thyroiditis on stable thyroid replacement therapy experienced a flare-up requiring more replacement. Her elevated baseline thyroid SUV_mean_ of 3.32 increased during treatment to 8.07 (Extended Data Fig. 7e,f). In other patients experiencing irAE ≥ grade 3 within the time frame of PET scans or thereafter, no higher ^89^ZED88082A uptake at baseline or during treatment occurred in organs of interest. This included two patients who developed diarrhea 4 and 14 days after the on-treatment CD8 PET. They were evaluated two days after the start of diarrhoea with colonoscopy and a colonic biopsy, which showed minor inflammation in both patients. They were later treated with steroids because of clinical suspicion of ICI-induced colitis.

**Extended Data Fig. 8:**
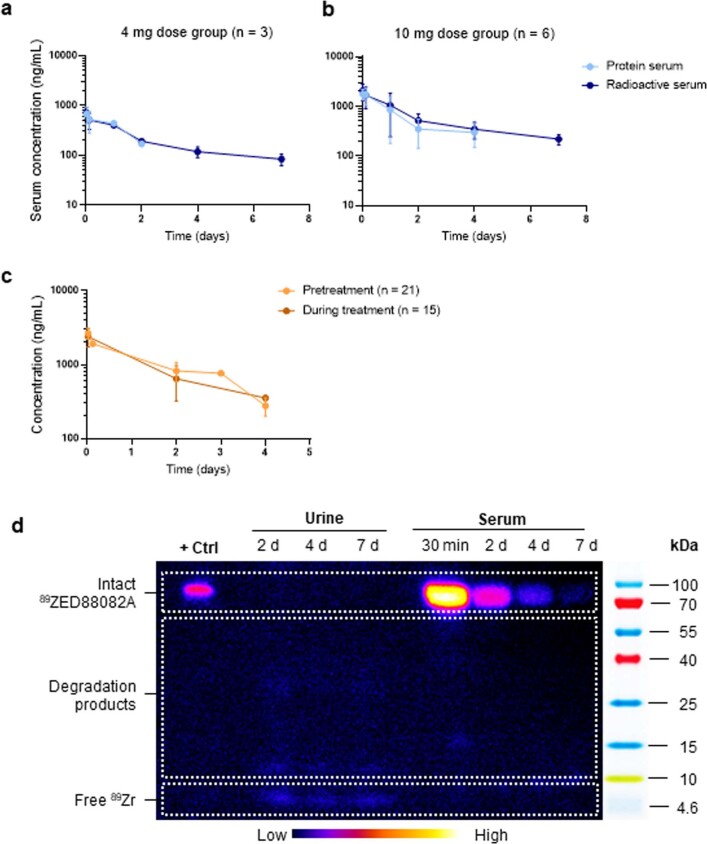
^89^ZED880082A/CED88004S pharmacokinetics and integrity. **a, b**, Time-concentration profiles (mean ± standard deviation) of ^89^ZED88082A/CED88004S serum protein and radioactivity following a single IV infusion of **a**, 4 mg (*n* = 3) and **b**, 10 mg (*n* = 6). **c**, Time-concentration profile (mean ± standard deviation) of ^89^ZED88082A/CED88004S pretreatment and during treatment in the presence of immune checkpoint inhibitor antibodies. **d**, Representative integrity assay (SDS-PAGE combined with autoradiography) of ^89^ZED880082A in urine and serum in samples drawn after 30 min and 2, 4, and 7 days after tracer injection (*n* = 3). Source data

In part A, serum ^89^ZED88082A/CED88004S protein levels were comparable within the same dose groups (Extended Data Fig. 8a,b). The estimated serum half-life of ^89^ZED88082A/CED88004S was 1.19 ± 0.33 days. Tracer pharmacokinetics were not influenced by ICI (Extended Data Fig. 8c). ^89^ZED88082A was intact in serum, while only low molecular weight components, including free ^89^Zr, were detectable in urine (Extended Data Fig. 8d). ^89^ZED88082A administration did not affect T cell, B cell and NK cell blood counts (Extended Data Fig. 3a).

No patient had endogenous antibody-drug antibodies (ADAs) before tracer injection (*n* = 31), 19% developed ADAs 28–50 days after the first (*n* = 5 out of 26) and 8% 18–38 days after the second tracer injection (*n* = 1 out of 12). One out of the 22 patients imaged twice (pre- and on treatment) developed ADAs after the first tracer injection. There was no apparent ADA effect on ^89^ZED88082A/CED88004S serum levels and imaging results.

## Discussion

A systemic characterization of the tumor microenvironment is critical for understanding an effective anticancer immune response following immunotherapies. This is a first-in-human study with the CD8-targeting antibody ^89^ZED88082A characterizing the CD8^+^ T cell biodistribution by PET imaging in patients with cancer at baseline and during ICI treatment. We demonstrated that the tracer is safe. Tracer uptake in tumor lesions correlated with CD8 IHC and autoradiography signal in those lesions. ^89^ZED88082A signal was conspicuous early on in the blood pool and kidneys as clearance organs, and in the spleen with extensive CD8 expression on the red pulp reticuloendothelial cells^18^. However, progressive uptake was evident only in CD8-rich tissues such as the lymph nodes, further supporting the tracer’s CD8 specificity.

Overall, high ^89^ZED88082A tumor uptake at baseline was associated with a better OS, concordant with findings from CD8 IHC in tissues from clinical ICI trials^6,19^. There was a major spatial heterogeneity within and between patients in ^89^ZED88082A uptake by their lesions. We took two approaches to verify whether potential differences in CD8 tracer uptake did reflect CD8-related tumor characteristics. First, we showed higher tracer uptake in dMMR than in pMMR tumors imaged before treatment, reflecting the higher CD8^+^ T cell infiltrate reported in dMMR tumors^20–24^. Second, we showed that tumor lesions biopsied and known by IHC to have a high T cell infiltrate (either ‘stromal’ or ‘inflamed’ phenotype) showed higher CD8 tracer uptake than the group with a low-T cell ‘desert’ phenotype. The ^89^ZED88082A uptake in a rim pattern in several tumors before and during treatment likely mirrors CD8^+^ T cell tumor infiltration referred to as the invasive margin^11,23,25^.

To improve insight into ICIs, their biodistribution has been studied with ^89^Zr-labeled anti-PD-1 and anti-PD-L1 antibodies^15,16,26,27^. In patients receiving atezolizumab, pretreatment ^89^Zr-atezolizumab tumor uptake predicted tumor response, PFS and OS, while PD-L1 expression assessed by IHC did not^15^. Similar observations were made for ^89^Zr-pembrolizumab imaging^16^. This demonstrates that T cells in tumor lesions as key mediators of immunotherapy can be evaluated by whole-body PET imaging. CD8 imaging was recently described in a small phase 1 study involving CD8 PET imaging at a single time point either before, during or after ICI or targeted therapy in 15 patients using different protein doses of the minibody ^89^Zr-Df-IAB22M2C^28^. The ^89^Zr-minibody was safe and accumulated in CD8^+^ rich tissues and tumor lesions of ten patients, supporting the CD8 PET approach.

Although we observed increasing signal in individual cases preceding a response, as also shown in some biopsy studies^9–11,29^, overall SUV_max_ changes on ^89^ZED88082A PET at 30 days after initiation of ICI did not correlate with best overall response when adjusted for volume changes. Intriguingly, we identified an enormous interlesional heterogeneity in tracer uptake on PET at 30 days in patients who responded. These findings indicate a remarkable spatio-temporal variability in systemic T cell dynamics as an antitumor immune response unfolds. Interestingly, similar results have been seen in a well-controlled mouse model using in situ fluorescent imaging of tumor cells and immune cells. Thus, a large variety in immunophenotype evolution was visualized even within individual mice of one model of the same seeded tumor cell line^30^. Moreover, in a human tumor fragment platform assay, PD-1 blockade resulted in different immune activation profiles among small tumor fragments derived from individual patient tumor lesions^31^. Together, our results underscore the importance of timing and characterization of all tumor lesions in comprehensively evaluating the tumor-immune status and therapy-induced pharmacodynamic effects.

Some tumor types display faster response kinetics to ICIs than others^32,33^. At 30 days, we captured a snapshot of patients and their lesions at different stages of their immune response, or lack thereof. Our results indicate that earlier imaging time points are warranted to capture CD8^+^ T cell dynamics that may be preceding the antitumor activity resulting in lesion shrinkage in these patients. Since various tumor types were included in our study, the numbers of individual tumor types enrolled were too small to define patient subset-specific CD8^+^ T cell kinetics. To fully understand and assess antitumor immunity induced by ICIs beyond what is feasible with localized tumor biopsies, it is essential to image T cell dynamics across lesions by whole-body evaluation over time. Because ^89^Zr has a relatively long half-life of 78.4 h, repeated PET imaging with ^89^Zr tracers ideally requires an interval of two weeks to avoid residual radioactivity and allow full clearance of the antibody. New small molecule tracers targeting CD8 and labeled with fluorine-18 may more readily allow sequential imaging time points, increasing the chance of capturing a more complete time course, to elucidate spatio-temporal changes in CD8^+^ T cells following initiation of immunotherapy^34^. For future studies, we envision also an earlier second imaging time point, namely within two weeks after starting ICI therapy, to capture pharmacodynamic changes before substantial tumor shrinkage.

Several issues challenged the interpretation of CD8 imaging changes following treatment. The uptake pattern changed rather than the magnitude of uptake in some tumor lesions, probably reflecting enhanced infiltration in a larger tumor volume. We expressed specific tumor uptake as SUV_max_, commonly used to measure specific uptake. However, this may not properly reflect heterogeneous uptake or a change in distribution pattern.

In addition, we detected CD8^+^ T cells in areas of nonmalignant inflammation, supporting the tracer’s ability to visualize inflammatory processes in any setting including ^89^ZED88082A PET changes during ICI treatment in a patient with Hashimoto’s thyroiditis, a disease with high lymphocyte involvement^35^. Therefore, CD8 PET may identify potential irAEs if patients are scanned in the relevant time frame. However, it should be noted that not all irAEs are driven by CD8^+^ T cells, and instead may involve multifactorial aetiologies including B cell, complement or auto-antibody driven mechanisms^36^. Thus, the potential relevance of CD8 PET in the characterization, identification and monitoring of irAEs will require further study and is currently limited to a single anecdote.

The tracer showed an organ-specific biodistribution in normal tissues without in vitro signs of cellular tracer internalization by immune cells. We cannot exclude that we also visualized CD8^+^ NK cells, but they are relatively rare and not likely to be confounding. Uptake in the spleen was conspicuous within the first hour postinjection, likely due to high perfusion and facile access of the tracer to high CD8 levels by littoral cells lining the red pulp sinusoids^15,18^. The higher spleen ^89^ZED88082A SUV_mean_ at 4 mg than at 10 mg likely reflects partial CD8 saturation at the 10 mg dose, due to containing more unlabeled CED88004S.

High bone marrow uptake early after injection, followed by a gradual decline in this densely vascularized space, is likely related to perfusion, while imaging at later time points likely reflects target-mediated ^89^ZED88082A binding to CD8^+^ T cells, which would be expected based on its role as a primary and secondary lymphoid organ and memory CD8^+^ T cell localization^37,38^. Moreover, we saw tracer uptake in the small intestine, likely showing CD8^+^ T cells in the gut-associated lymphoid tissue, such as the Peyer’s patches within the gut mucosa^39,40^. High tracer uptake in these tissues matched sites of CD8 protein expression reported in the Protein Atlas^41^, although these comparisons cannot be exact due to the relatively young and healthy sources of tissues in the atlas, and the relative complexity of delivering antibody tracer to the CD8 target in living subjects. Tracer signals in liver, renal cortex, urine and large bowel probably reflected tracer clearance and metabolism rather than target-mediated binding. The renal cortex showed a persistent high radioactive signal irrespective of decreasing blood pool levels. This is presumably due to renal tracer clearance followed by resorption and catabolism with residualization of intracellular charged metal chelate catabolites such as lysine-DFO-Zr-binding proteins. This is a known phenomenon for small molecules and antibody fragments^42,43^.

Serial, whole-body characterization of CD8^+^ T cells has several potential applications in clinical research. One application is to more fully characterize the pretreatment CD8^+^ T cell tumor infiltration, which may function as a predictive biomarker for subsequent response to a particular immunotherapy (for example, ICIs). Furthermore, serial CD8 PET imaging has the potential to characterize treatment-emergent pharmacodynamic changes following new immunotherapies or combinations of agents, and may therefore prove useful in guiding their clinical development. ^89^ZED88082A PET may also be helpful to guide tumor biopsies to improve the chance of obtaining a tumor sample with high CD8^+^ T cell infiltration. Ultimately, CD8 PET has the potential to become a clinical decision support tool to individualize immunotherapeutic approaches in patients. Describing and accepting the huge spatial and temporal heterogeneity of CD8^+^ T cells is critical towards a more individualized treatment approach in the future. However, the generation of much larger CD8 PET imaging data sets and correlation with clinical outcomes will be needed to assess whether CD8 PET can guide treatment decisions.

In conclusion, ^89^ZED88082A PET specifically visualizes CD8 in vivo, offering the opportunity to assess whole-body CD8^+^ T cell distribution, not obtainable with a single-lesion biopsy. We demonstrated that CD8^+^ T cell presence in tumor lesions imaged before ICI could be predictive for OS, highlighting the potential of CD8 imaging as a predictive biomarker to personalize treatment for patients. The dynamics of intratumoral CD8 expression during ICI exposure is more complex and nuanced than previously reported and differs between patients and lesions in the same patient. To properly evaluate tumor-immune status, timing and evaluation across lesions are crucial. Our results provide a strong rationale to characterize the tumor-immune microenvironment using new imaging technologies.

## Methods

### Study design

This single-center imaging study comprised parts A and B. Eligible patients for part A or B1 had a histologically confirmed locally advanced or metastatic cancer, whom, in the investigator’s opinion, based on available clinical data, may benefit from anti-PD-L1 antibody treatment, and had disease progression during or following first-line standard-of-care therapy. In part B2, patients with melanoma eligible for standard-of-care ICIs could participate. Eligible patients had measurable disease according to RECIST1.1, were amenable to a tumor biopsy, were ≥18 years of age and had an Eastern Cooperative Oncology Group performance status of 0–1, life expectancy ≥12 weeks, and adequate hematologic and end-organ function. Patients with concomitant or historical conditions or medication use that could compromise their safety or interpretation of study results were excluded.

The study was performed with a companion treatment study with atezolizumab for parts A and B1 at the University Medical Center Groningen (NCT02478099). All patients provided written informed consent for the imaging and, if applicable, the treatment study. The studies were approved by the Medical Ethical Committee of the University Medical Center Groningen and the Central Committee on Research Involving Human Subjects.

Patients received zirconium-89-labeled CED88004S (^89^ZED88082A) with unlabeled DFO-conjugated one-armed antibody CED88004S intravenously as two consecutive boluses. In dose-finding part A, patients received tracer injection before atezolizumab treatment, consisting of 37 MBq (1.2–1.5 mg) ^89^ZED88082A with additional unlabeled CED88004S until a total protein dose of 4 mg (*n* = 3) or 10 mg (*n* = 6). The unlabeled dose was varied to allow for adequate tracer blood pool availability, comparable with earlier studies^44^. The first two patients at each dose level during dose-finding were hospitalized overnight for safety monitoring. After tracer injection, PET scans were performed at 1 h, and days 2, 4 and 7, followed by a biopsy of a tumor lesion identified before the PET scan. In part B, patients received tracer and PET scans before and early during ICI cycle 2 (~30 days), with optimal protein dose and PET scan schedule based on part A. After baseline PET scans and tumor biopsy, patients from parts A and B1 received 1,200 mg atezolizumab intravenously every three weeks. Patients with melanoma received standard-of-care immunotherapy. After part A was closed, part B was opened. Cohort assignment was in the order of enrollment.

### ^89^ZED88082A tracer and PET procedures

Unlabeled, DFO-conjugated one-armed antibody CED88004S, provided by Genentech Inc., was radiolabelled with ^89^Zr-oxalate (^89^ZED88082A) according to good manufacturing practice guidelines^17^. Based on stability testing, ^89^ZED88082A shelf-life was defined as 96 h at 2–8 °C in the vial and an additional 4 h at room temperature in the syringe. See Supplementary Table 1 for release specifications.

PET scans were acquired with low-dose CT for attenuation correction and anatomic localization, with a Biograph mCT 64-slice, Biograph mCT 40-slice or Biograph Vision (128-slice) PET/CT camera (all Siemens, software versions VG70B/VG70C/VG60C/CG70C/VG76A/VG80A). PET scan acquisition consisted of total body mode (skull to feet) up to 15 bed positions depending on the patient’s length (Biograph mCTs) or total four passes (Vision). Baseline and repeated PET scans in cohort B were performed on the same machine. According to harmonization procedures, PET reconstruction was compatible with the EARL1 PET/CT accreditation and European Association of Nuclear Medicine guidelines^45^. PET images were visually evaluated (Syngo.via, version VB_40.02), and analyzed using the Accurate tool^46^ (versions .08072019, .22042020 and .14082020). Spherical VOIs were drawn around tumor lesions ≥1 cm and in organs of interest to assess the tracer biodistribution. Tumor lesions ≥1 cm in diameter were identified at baseline on diagnostic CT or MRI or via clinical evaluation for (sub)cutaneous lesions, and VOIs were delineated manually for PET images analysis on tracer uptake. Tracer uptake in nonmalignant lymph nodes was qualitatively assessed and quantified on the PET scan images in the cervical, axillary and inguinal regions. Tracer uptake in Waldeyer’s ring was omitted after previous tonsillectomy and/or adenoidectomy, and no visual uptake on PET. All PET scans were visually evaluated for unexpected tracer uptake.

SUV was calculated using bodyweight, net injected radioactivity dose and radioactivity within a VOI. All SUVs reported are at 10 mg on day 2 postinjection unless specified otherwise.

### Tumor tissue analyses

Tumor biopsies were performed within ten days after tracer injection and within four days after the last PET scan. Whole tissue blocks of formalin-fixed, paraffin-embedded (FFPE) biopsy samples were analyzed with autoradiography. Thereafter, 4 µm sections were stained with haematoxylin and eosin, and CD8 was IHC stained with the mouse CD8 monoclonal antibody C4/144B (DAKO/Agilent). IHC images were captured with Philips Intellisite Pathology solution v.3.2. If baseline biopsy lacked, archival tumor tissue was studied. Tissue sections that did not contain tumor were excluded from IHC/PET analyses.

CD8 expression was determined by a pathologist (H.K.) blinded for treatment outcome, and CD8^+^ T cell infiltration was described as desert, stromal or inflamed phenotype^47,48^. For stromal or inflamed tumor tissues, CD8^+^ T cell density was assessed as 1 (minor), 2 (intermediate) or 3 (high) as a subjective estimate of average density considering the entire tumor area to address intratumoral heterogeneity. Representative examples in Extended Data Fig. 4a.

Whole FFPE tumor tissue blocks were exposed for six to eight days to a multipurpose or multisensitive phosphor storage plate (PerkinElmer). Exposures were captured using a Cyclone phosphor imager. To correlate ^89^ZED88082A uptake with the spatial patterning and intensity of CD8 expression, autoradiography images were scaled and registered to IHC images using manually selected control points and an affine transformation for 16 tumor slides. IHC CD8 expression was expressed as the percentage of CD8^+^ positive pixels across the manually defined region of interest (ROI) specific to tumor including tumor-associated stroma per slide (excluding normal stroma and background tissue), thus CD8 IHC positive pixels/all pixels of the tumor area. ^89^ZED88082A tissue uptake was measured as digital autoradiograph signal for the ROI corrected by background subtraction on a per slide basis. Decay correction was applied to adjust for differences in the timing of sample scanning after injection. Slide-level analyses served to evaluate the tracer’s ability to distinguish specimens of relatively high and low CD8 expression (pixel-based). For each slide, average IHC percent positivity and autoradiographic tracer intensity were computed globally and locally using overlapping square tiles of varying sizes (100 × 100 pixels, 400 × 400 pixels, 1,000 × 1,000 pixels to 8,000 × 8,000 pixels). Only tiles with ≥25% overlap with tumor ROI were included. Image scaling, registration and summarization were executed using MATLAB (Mathworks). Decay correction was applied to autoradiography tracer intensities to adjust for differences in the timing of sample collection after injection.

Tumors were considered dMMR if at least one of the following criteria was applicable^49^: tumor showed loss of ≥1 MMR proteins MLH1, MSH2, MSH6 or PMS2, assessed by IHC; DNA analysis showed high microsatellite instability; patients with known germline mutation in MMR genes in the context of hereditary nonpolyposis colorectal cancer syndrome. If unavailable at study entry, MMR protein status was assessed immunohistochemically on (archival) tumor tissue. If the result was equivocal, DNA analysis for microsatellite instability was performed.

### Laboratory analyses

In part A, blood samples for pharmacokinetics were collected before injection and at 30 min, 3 h, one or two days, four days and seven days postinjection; in part B, before and 30 min postinjection and at day of PET scan, for pretreatment as well as on-treatment PET series. Tracer levels were analyzed with an ELISA of serum ^89^ZED88082A/CED88004S and with serum ^89^Zr-radioactivity measurements. Clinical samples, assay calibrators and controls were captured on a microtiter plate using a rabbit monoclonal antibody to CED88004S. For detection, a biotin-conjugated anti-human IgG followed by a streptavidin-horseradish peroxidase incubation and a colorimetric reaction were used. The calibration curve range is 149 to 2,500 ng ml^−1^. Half-life of ^89^ZED88082A/CED88004S was estimated by standard noncompartmental analysis using Phoenix WinNonlin (Certara Inc., v.6.4) and is presented as (average ± standard deviation).

Serum samples, drawn before the first and second tracer injection and 30 days after the last injection, were analyzed for ADAs using a bridging ELISA assay with a relative sensitivity of 22 ng ml^−1^. ADA-positive subjects were defined as those who screened negative for ADAs at baseline and had ADAs following ^89^ZED88082A/CED88004S administration (positive in the ADA confirmatory assay).

Blood was collected in sodium heparin tubes before and two to seven days after the first tracer injection for peripheral blood lymphocyte analyses. Peripheral blood mononuclear cells (PBMCs) were isolated by Ficoll gradient centrifugation in LeucoSep-tubes (Greiner Bio-One) and resuspended in freeze medium using CTL-Cryo ABC Media Kit (CTL Europe GmbH). Cryovials were stored in liquid nitrogen until analysis. T, B and NK cell enumeration was determined flow cytometrically.

^89^ZED88082A stability was studied in serum and urine collected at days 0, 4 and 7, with sodium dodecyl sulfate-polyacrylamide gel electrophoresis^50^. Intact ^89^ZED88082A and radioactive degradation products were detected autoradiographically by exposing gels to a multipurpose phosphor plate (PerkinElmer) overnight at −20 °C. Exposures were captured using a Cyclone phosphor imager. Images were analyzed using ImageJ (v.1.52p).

For tracer CD8-receptor mediated binding and internalization analysis, PBMCs were prepared from healthy blood donor buffy coats (Sanquin) with appropriate informed consent, by centrifugation in LeucoSep-tubes (Greiner Bio-One). Unstimulated PBMCs were diluted to 1 million cells ml^−1^ in phosphate-buffered saline containing 2% fetal calf serum (FACS buffer). CED88004S was diluted in FACS buffer to 20 µg ml^−1^ and incubated with the PBMCs for 1 or 2 h at 37 °C. CED88004S binding to CD8 and subsequent cellular internalization in PBMCs was determined flow cytometrically^51^. For characterization of CD3-positive cell populations, peridinin chlorophyll protein complex-cyanine5.5 (PerCP/Cy5.5)-conjugated mouse anti-human CD3 monoclonal antibody clone OKT3 (Thermofisher Scientific; 45-0037-42) was used. Membrane-bound CED88004S was detected using allophycocyanin-conjugated donkey anti-human IgG F(ab′)2 fragment (Jackson ImmunoResearch Laboratories; 709-136-149) within the total PBMC population (Extended Data Fig. 7, blue) or CD3-positive cell population (Extended Data Fig. 7, red). Samples were analyzed on a BD FACS Verse flow cytometer (BD Biosciences, Supplementary Fig. 2). Samples were measured in duplicate, corrected for background fluorescence and nonspecific antibody binding. Data analysis was performed with FlowJo v.10 (Tree Star). The presence of surface receptors was expressed as mean fluorescent intensity.

### Clinical outcomes and CT analysis

Safety was assessed according to the common terminology criteria for AEs of the National Cancer Institute, v.4.0. Tracer-related AEs were collected from the first tracer injection until 30 days after the last tracer injection. For analyses of tracer uptake and immune-related ICI-induced toxicity, PET scans were evaluated for organs of interest in patients who experienced irAEs grade ≥3.

Before therapy, patients had a contrast-enhanced diagnostic CT-chest-abdomen and brain CT or MRI. According to RECIST1.1 or iRECIST if applicable^52,53^, response evaluation was performed every six weeks during atezolizumab treatment or 12 weeks in patients with melanoma. The sum of longest diameter (SLD) according to RECIST is the sum of the maximal diameter of target lesions, with short axis in the case of lymph nodes; best overall response is the most favorable response confirmed by a consecutive assessment. PFS and OS were determined from the first treatment dose until disease progression, or death from any cause, for PFS, whichever occurred first. For PFS, data from subjects without disease progression and death were censored at the date of last tumor assessment, or, if no tumor assessments were made after the baseline visit, at the date of first treatment plus one day. To interpret tumor-rim uptake, tumor necrosis was defined as 10–30 Hounsfield Units in portal venous phase on CT.

### Statistical analysis

We used standard descriptive statistics to describe the distribution of various characteristics, including ^89^ZED88082A uptake.

As a general approach, the relation between ^89^ZED88082A uptake in tumor lesions and in normal tissues with various determinants (time since tracer injection, protein dose level, tumor lesion organ location, MMR status, immune phenotype, best overall response and ICI treatment status) were assessed using linear mixed models to account for repeated measurements within patients using random intercepts and, if applicable, within tumor lesions using additional random intercepts nested within patients. For tumor lesions and normal lymph nodes and tonsils, we used SUV_max_ as the ^89^ZED88082A-uptake measure, which was log-transformed in the analyses to account for its right-skewed distribution, and results were subsequently back-transformed to obtain estimates of geometric means and percent differences. The ^89^ZED88082A uptake in other normal tissues was expressed as SUV_mean_, which was analyzed without transformation, yielding estimates of means and mean differences. To obtain ^89^ZED88082A-uptake estimates, we fitted the linear mixed models under restricted maximum likelihood and used Satterthwaite degrees of freedom to obtain 95% CIs and Wald *P* values. In addition, we obtained likelihood ratio *P* values from models fitted under maximum likelihood. A trend test for the relation between best overall response and tumor ^89^ZED88082A uptake was obtained by analyzing best overall response categories as a numerical variable (with PD, SD, PR and CR expressed as 0, 1, 2 and 3, respectively).

Using data from study part A, postinjection time-uptake curves were fitted using postinjection imaging time point both categorically and continuously, selecting the best curve-fit for the latter from a linear, a log-linear or a quadratic fit using the Akaike’s Information Criterion (under maximum likelihood). Protein dose level varied in part A and was included in these models as a main effect and using an interaction term with postinjection time. As the shape of the time-uptake curves did not substantially depend on protein dose level, the main results of these analyses included protein dose level as a main effect only, and the resulting estimates from these models were projected at the 10 mg protein dose level. This was the protein dose taken forward towards part B of the study. All other analyses were performed in patients receiving a 10 mg protein dose level.

Regarding ^89^ZED88082A-uptake change during ICI therapy, we defined the on-treatment measurement as the actual time between start of ICI therapy and the on-treatment ^89^ZED88082A PET assessment to account for variation between patients in the timing of the PET scan (pretreatment assessment assumed to represent the situation before start of ICI and therefore the time between pretreatment assessment and start of ICI was set at zero days for this analysis). The results are expressed as changes in ^89^ZED88082A uptake per week of ICI therapy, also summarized as expected values at 30 days of ICI therapy which was the median time point across patients. To assess whether the ^89^ZED88082A-uptake change depended on ICI treatment response, we used interaction terms between treatment status and best overall response, separating patients into a PD and a non-PD group due to the limited number of patients prohibiting more detail.

For tumor uptake change during ICI therapy, we attempted to account for possible shrinkage of individual tumor lesions leading to an underestimation of actual uptake due to partial volume effects in a data-driven way. For this, we first assessed the relation between CT-measured tumor lesion volume (based on two orthogonal measurements assuming an oblate spheroid shape) and geometric mean ^89^ZED88082A uptake in 238 lesions from 34 patients (all with 10 mg protein dose) only using the treatment-naive measurements and using a 5-knot restricted cubic spline. This showed that lesions <2 cm^3^ exhibited a decrease in the measured geometric mean ^89^ZED88082A uptake with decreasing volume (as expected), while for lesions between 2 and 65 cm^3^ (the 95th percentile), there was no relation between volume and ^89^ZED88082A uptake. Using this observed relation between volume and geometric mean ^89^ZED88082A uptake in the pretreatment data, we next expressed the observed ^89^ZED88082A uptake of individual lesions as the absolute difference compared to the expected geometric mean uptake of lesions of identical volume based on the restricted cubic spline curve, for the pretreatment and on-treatment measurements, and then added to this difference between observed and expected ^89^ZED88082A uptake the expected geometric mean pretreatment uptake of lesions of 5 cm^3^ (quite arbitrarily chosen within the volume range without an observed relation with pretreatment ^89^ZED88082A uptake). To account for the time period between on-treatment ^89^ZED88082A PET scan and first CT for response evaluation, we linearly interpolated the volume change between baseline and the first on-treatment response CT to obtain an expected lesion volume at the timing of the ^89^ZED88082A PET. The resulting tumor-volume-adjusted ^89^ZED88082A-uptake values can be interpreted as the absolute difference in ^89^ZED88082A uptake compared to treatment-naive lesions of the same size, projected for all lesions towards a lesion volume of 5 cm^3^ (that is, resulting in an estimation of the amount of increased or decreased ^89^ZED88082A uptake compared to an average lesion of 5 cm^3^). Finally, the resulting volume-adjusted ^89^ZED88082A-uptake variable was analyzed for its relationship with uptake change during treatment and treatment response status similarly as the actual measured ^89^ZED88082A uptake. An underlying assumption of the above approach is that the empirically observed relation between volume and ^89^ZED88082A uptake in the pretreatment data accurately captures the true partial volume effect phenomenon. We specifically chose this approach beyond merely adjusting the analyses for estimated tumor volume directly, because of the potential mixing of on-treatment effects between volume and ^89^ZED88082A uptake.

To investigate the relation between pretreatment ^89^ZED88082A uptake and PFS and OS, ^89^ZED88082A uptake was expressed as geometric mean SUV_max_ per patient and then analyzed both categorically (based on a median-split across patients) and continuously (expressed per population standard deviation—the entire per patient geometric mean SUV_max_ distribution encompasses approximately six times this population standard deviation). Given the small dataset, we specifically refrained from exploring potentially more optimal cut-off levels than the predefined median split to avoid overoptimistic results. Similarly, for the continuous analyses, we assumed (log)linearity and refrained from exploring other functional forms. We used Kaplan–Meier curves and log-rank tests and obtained hazard ratios using Cox regression models with Firth’s penalization to account for small sample bias. The above statistical analyses were performed using R v.4.1.1 for macOS, particularly using the lmer function for linear mixed models (lme4 v.1.1-27.1, lmerTest v.3.1-3), coxphf for Cox models (coxphf v.1.13.1) and rcspline.eval for restricted cubic splines (rms v.6.2-0). All *P* values are based on two-sided statistical tests without correction for multiple testing.

Slide-level correlation between autoradiography and IHC was assessed by Kendall’s rank-based correlation. Subslide (tile) level analyses were also performed to evaluate the ability of the tracer to identify localized regions of CD8 positivity within individual biopsy samples. For tile-level analyses, autoradiography images were scaled and aligned to CD8 IHC images using manually selected control points and an affine transformation. Local average autoradiography and IHC measurements for each slide were computed in overlapping tiles of varying sizes. Association between autoradiography and IHC was assessed using Kendall’s rank-based correlation within samples and after pooling across samples. Within the sample, tile-level correlations were calculated at each tile size only for samples with ≥6 tiles as the variance of estimated correlations is high at smaller sample sizes.

### Reporting summary

Further information on research design is available in the Nature Portfolio Reporting Summary linked to this article.

## Online content

Any methods, additional references, Nature Portfolio reporting summaries, source data, extended data, supplementary information, acknowledgements, peer review information; details of author contributions and competing interests; and statements of data and code availability are available at 10.1038/s41591-022-02084-8.

### Supplementary information

Supplementary Information Supplementary Figs. 1 and 2 and Table 1.

Reporting Summary

Supplementary Video Whole-body visualization of a representative ^89^ZED88082A PET scan day 2 with total protein dose 10 mg (maximum intensity projection). Tracer uptake is visible in tumor lesions in the right breast and liver, and normal lymphoid tissues including spleen, lymph nodes, Waldeyer’s ring and small intestine.

### Source data

Source Data Extended Data Fig. 8d Unprocessed SDS–PAGE combined with autoradiography.

Source Data Fig. 3a Unprocessed tumor autoradiography image.

## Figures and Tables

**Extended Data Table 1:**
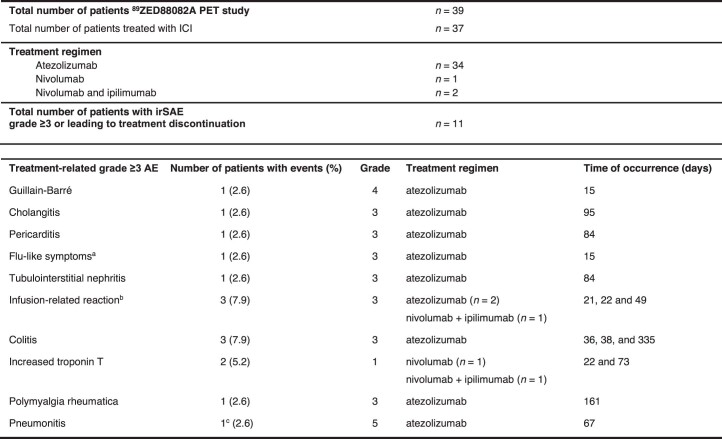
Summary of immune-related serious adverse events (irSAEs) grade ≥ 3 or leading to treatment discontinuation

All adverse events were scored according to CTCAE v4.0. Only treatment-related serious adverse events are listed here. Time of occurrence after initiation of ICI therapy is provided in days. ^a^Characterized by fever, headache and/or myalgia, which did not occur during infusion, and infection-related causes were excluded by diagnostics. ^b^Characterized by fever or chills during infusion. ^c^This patient experienced a grade 3 colitis after 2 cycles atezolizumab, followed by a pneumonitis after 24 days atezolizumab postponement, and died due to respiratory insufficiency.

## Data Availability

The study protocol and clinical details of the cases and laboratory data, restricted to nonidentifying data owing to privacy concerns, can be requested from the corresponding author, who will handle all requests. Genentech developed and owns the intellectual property rights pertaining to CED88004S. Source data are provided with this paper. All other materials are readily available from the authors or commercial sources.

## References

[1] Hodi FS (2018). Nivolumab plus ipilimumab or nivolumab alone versus ipilimumab alone in advanced melanoma (CheckMate 067): 4-year outcomes of a multicentre, randomised, phase 3 trial. Lancet Oncol..

[2] Vaddepally RK (2020). Review of indications of FDA-approved immune checkpoint inhibitors per NCCN guidelines with the level of evidence. Cancers.

[3] Chang E (2021). Systematic review of PD-1/PD-L1 inhibitors in oncology: from personalized medicine to public health. Oncologist.

[4] Havel JJ, Chowell D, Chan TA (2019). The evolving landscape of biomarkers for checkpoint inhibitor immunotherapy. Nat. Rev. Cancer.

[5] Herbst RS (2014). Predictive correlates of response to the anti-PD-L1 antibody MPDL3280A in cancer patients. Nature.

[6] Lee JS, Ruppin E (2019). Multiomics prediction of response rates to therapies to inhibit programmed cell death 1 and programmed cell death 1 ligand 1. JAMA Oncol..

[7] Wong PF (2019). Multiplex quantitative analysis of tumor-infiltrating lymphocytes and immunotherapy outcome in metastatic melanoma. Clin. Cancer Res..

[8] Ribas A (2009). Intratumoral immune cell infiltrates, FoxP3, and indoleamine 2,3-dioxygenase in patients with melanoma undergoing CTLA4 blockade. Clin. Cancer Res..

[9] Edwards J (2018). CD103+ tumor-resident CD8+ T cells are associated with improved survival in immunotherapy-naïve melanoma patients and expand significantly during anti-PD-1 treatment. Clin. Cancer Res..

[10] Chen PL (2016). Analysis of immune signatures in longitudinal tumor samples yields insight into biomarkers of response and mechanisms of resistance to immune checkpoint blockade. Cancer Discov..

[11] Tumeh PC (2014). PD-1 blockade induces responses by inhibiting adaptive immune resistance. Nature.

[12] Litchfield K (2020). Representative sequencing: unbiased sampling of solid tumor tissue. Cell Rep..

[13] Jiménez-Sánchez A (2017). Heterogeneous tumor-immune microenvironments among differentially growing metastases in an ovarian cancer patient. Cell.

[14] de Vries EGE (2019). Integrating molecular nuclear imaging in clinical research to improve anticancer therapy. Nat. Rev. Clin. Oncol..

[15] Bensch F (2018). ^89^Zr-atezolizumab imaging as a non-invasive approach to assess clinical response to PD-L1 blockade in cancer. Nat. Med..

[16] Kok, I. C. et al. ^89^Zr-pembrolizumab imaging as a non-invasive approach to assess clinical response to PD-1 blockade in cancer. *Ann. Oncol*. **33**, 80–88 (2022). 10.1016/j.annonc.2021.10.213 34736925

[17] Gill H (2020). The production, quality control, and characterization of ZED8, a CD8-specific ^89^Zr-labeled immuno-PET clinical imaging agent. AAPS J..

[18] Ogembo JG (2012). SIRPα/CD172a and FHOD1 are unique markers of littoral cells, a recently evolved major cell population of red pulp of human spleen. J. Immunol..

[19] Li F (2021). The association between CD8+ tumor-infiltrating lymphocytes and the clinical outcome of cancer immunotherapy: a systematic review and meta-analysis. EClinicalMedicine.

[20] Le DT (2015). PD-1 blockade in tumors with mismatch-repair deficiency. N. Engl. J. Med..

[21] Prall F (2004). Prognostic role of CD8+ tumor-infiltrating lymphocytes in stage III colorectal cancer with and without microsatellite instability. Hum. Pathol..

[22] Millen R (2020). CD8+ tumor-infiltrating lymphocytes within the primary tumor of patients with synchronous de novo metastatic colorectal carcinoma do not track with survival. Clin. Transl. Immunol..

[23] Yoon HH (2019). Intertumoral heterogeneity of CD3^+^ and CD8^+^ T-cell densities in the microenvironment of DNA mismatch-repair–deficient colon cancers: implications for prognosis. Clin. Cancer Res..

[24] Narayanan S (2019). Tumor infiltrating lymphocytes and macrophages improve survival in microsatellite unstable colorectal cancer. Sci. Rep..

[25] Gallon J, Bruni D (2019). Approaches to treat immune hot, altered and cold tumours with combination immunotherapies. Nat. Rev. Drug Discov..

[26] Niemeijer AN (2018). Whole body PD-1 and PD-L1 positron emission tomography in patients with non-small-cell lung cancer. Nat. Commun..

[27] van de Donk PP (2020). Molecular imaging biomarkers for immune checkpoint inhibitor therapy. Theranostics.

[28] Farwell MD (2022). CD8-targeted PET imaging of tumor infiltrating T cells in patients with cancer: a phase I first-in-human study of ^89^Zr-Df-IAB22M2C, a radiolabeled anti-CD8 minibody. J. Nucl. Med..

[29] Ribas A (2016). PD-1 Blockade expands intratumoral memory T cells. Cancer Immunol. Res..

[30] Ortiz-Muñoz, G. et al. Surveillance of in situ tumor arrays reveals early environmental control of cancer immunity. Preprint at *bioRxiv*10.1101/2021.05.27.445482 (2021).

[31] Voabil P (2021). An ex vivo tumor fragment platform to dissect response to PD-1 blockade in cancer. Nat. Med..

[32] Borcoman E (2019). Novel patterns of response under immunotherapy. Ann. Oncol..

[33] Hamid O (2019). Five-year survival outcomes for patients with advanced melanoma treated with pembrolizumab in KEYNOTE-001. Ann. Oncol..

[34] Rosenberg A (2021). Development of a fully automated method for radiosynthesis of fluorine-18 labeled CD8 PCC radiotracers. J. Nucl. Med..

[35] Liblau RS (2012). Autoreactive CD8 T cells in organ-specific autoimmunity: emerging targets for therapeutic intervention. Immunity.

[36] Postow MA (2018). Immune-related adverse events associated with immune checkpoint blockade. N. Engl. J. Med..

[37] Bonomo A (2016). A T cell view of the bone marrow. Front. Immunol..

[38] Shin SS (1992). lmmunoarchitecture of normal human bone marrow: a study of frozen and fixed tissue sections. Hum. Pathol..

[39] Sathaliyawala T (2013). Distribution and compartmentalization of human circulating and tissue-resident memory T cell subsets. Immunity.

[40] Heel KA (1997). Review: Peyer’s patches. J. Gastroenterol. Hepatol..

[41] Uhlén, M. et al. Tissue-based map of the human proteome. *Science* **347**, 1260419 (2015). 10.1126/science.1260419 25613900

[42] Behr TM (1995). Reduction of the renal uptake of radiolabeled monoclonal antibody fragments by cationic amino acids and their derivatives. Cancer Res..

[43] Akizawa H (2008). Renal uptake and metabolism of radiopharmaceuticals derived from peptides and proteins. Adv. Drug Deliv. Rev..

[44] Bensch F (2018). Comparative biodistribution analysis across four different ^89^Zr-monoclonal antibody tracers: the first step towards an imaging warehouse. Theranostics.

[45] Makris NE (2014). Multicenter harmonization of ^89^Zr PET/CT performance. J. Nucl. Med..

[46] Boellaard R (2018). Quantitative oncology molecular analysis suite: ACCURATE. J. Nucl. Med..

[47] Hegde PS (2016). The where, the when, and the how of immune monitoring for cancer immunotherapies in the era of checkpoint inhibition. Clin. Cancer Res..

[48] Mariathasan S (2018). TGFβ attenuates tumour response to PD-L1 blockade by contributing to exclusion of T cells. Nature.

[49] Weissman SM (2011). Genetic counseling considerations in the evaluation of families for Lynch syndrome: a review. J. Genet. Couns..

[50] Giesen D (2020). Probody therapeutic design of ^89^Zr-CX-072 promotes accumulation in PD-L1–expressing tumors compared to normal murine lymphoid tissue. Clin. Cancer Res..

[51] Kol A (2017). ADCC responses and blocking of EGFR-mediated signaling and cell growth by combining the anti-EGFR antibodies imgatuzumab and cetuximab in NSCLC cells. Oncotarget.

[52] Eisenhauer EA (2009). New response evaluation criteria in solid tumours: revised RECIST guideline (version 1.1). Eur. J. Cancer.

[53] Seymour L (2019). iRECIST: guidelines for response criteria for use in trials testing immunotherapeutics. Lancet Oncol..

