# Supplementary material for: Whole-body CD8+ T cell visualization before and during cancer immunotherapy: a phase 1/2 trial
Source: Nat Med. 2022 Dec 5;28(12):2601–10. doi: 10.1038/s41591-022-02084-8 (PMC9800278; doi:10.1038/s41591-022-02084-8)
Supplement: Supplementary file 1 — Supplementary Figs. 1 and 2 and Table 1. [file 41591_2022_2084_MOESM1_ESM.pdf]

nature medicine

Article

https://doi.org/10.1038/s41591-022-02084-8

Whole-body CD8$^+$ T cell visualization before and during cancer immunotherapy: a phase 1/2 trial

In the format provided by the authors and unedited

Supplementary Information

Whole-body CD8$^+$ T-cell visualization before and during cancer immunotherapy: a phase 1/2 trial

Kist de Ruijter, et al.

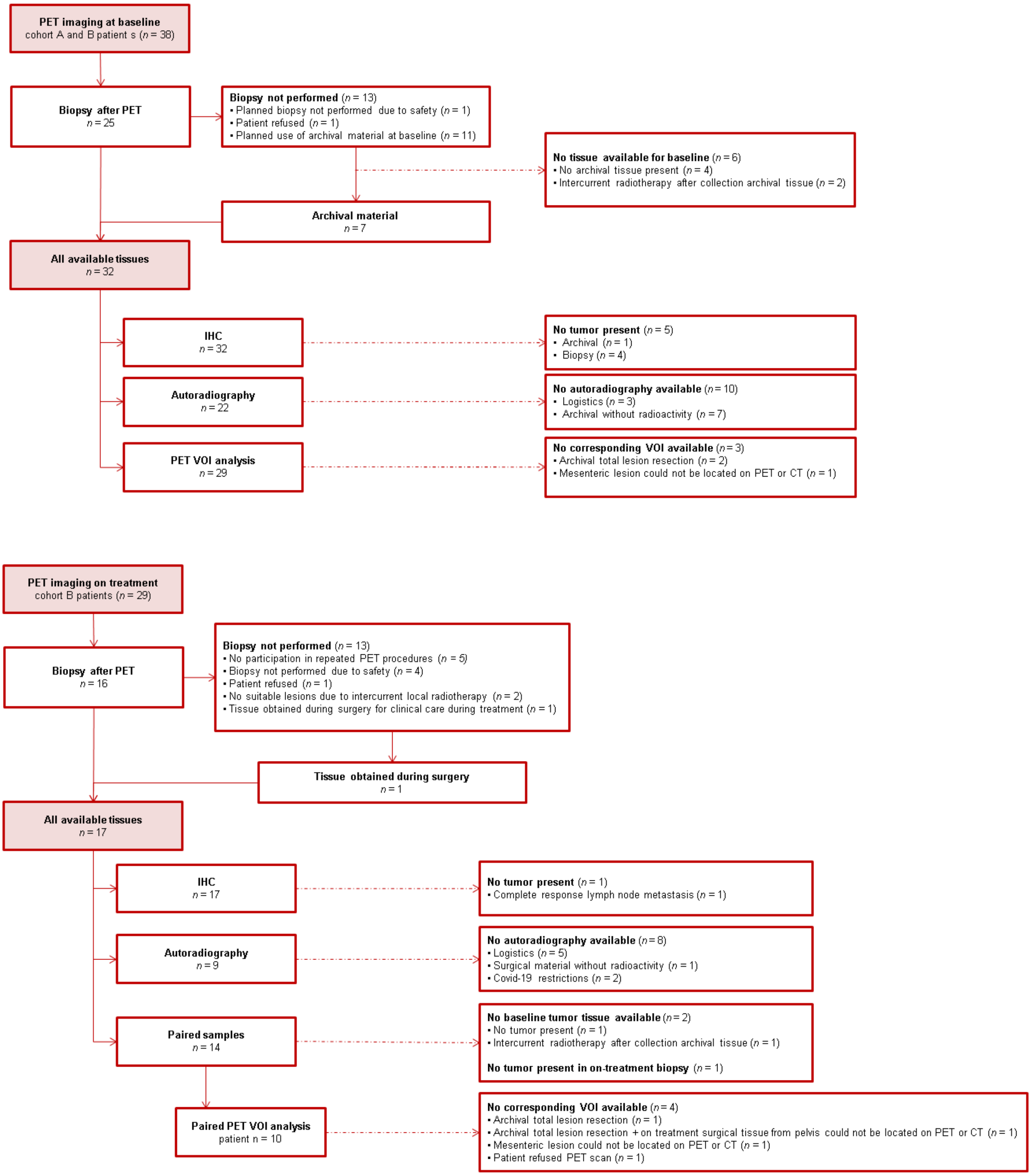

Supplementary information Figure S1 | Flow charts indicate the number of tumour tissues available for analysis at baseline and during treatment. IHC, immunohistochemistry. VOI, volume of interest.

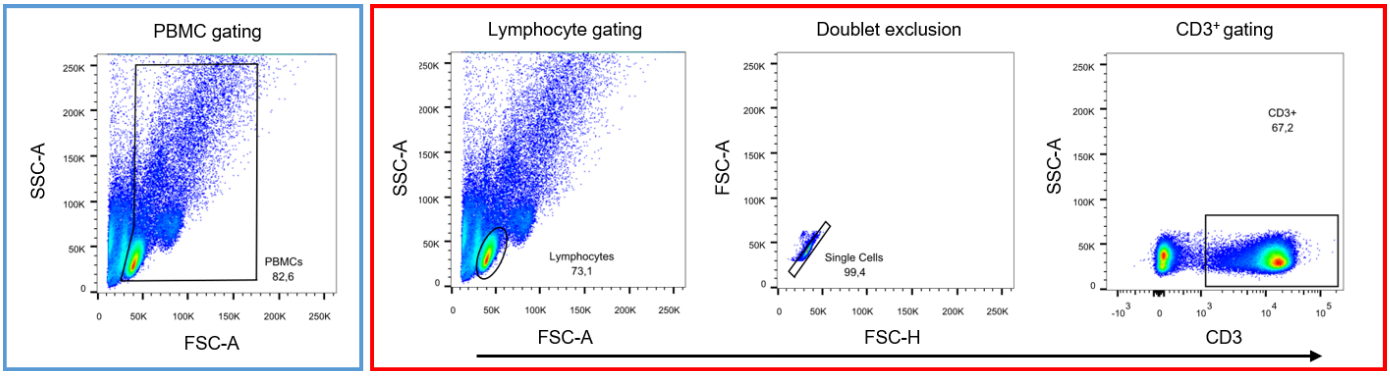

Supplementary information Figure S2 | Flow cytometry gating strategy used in internalization experiments, supplementary to Extended Data Figure 7. Peripheral blood mononuclear cells (PBMCs) were gated in a forward scatter (FSC) versus side scatter (SSC) dot plot. Lymphocytes were gated in an FSC versus SSC dot plot, doublets were excluded by plotting FSC height (FSC-H) versus area (FSC-A). CD3 positive T cells were gated on the anti-human peridinin chlorophyll protein complex-cyanine5.5 (PerCP/Cy5.5)-CD3 staining. Binding/internalization of CED88004S was detected using an anti-human allophycocyanin-IgG F(ab')2 fragment within the total PBMC population (blue) or CD3-positive cell population (red). At least 10,000 events were measured within the $CD3^+$ cell population. CED88004S membrane binding is expressed as mean fluorescent intensity (MFI), and no cell sorting was applied. Samples were measured in duplicate and corrected for background fluorescence and non-specific antibody binding.

Supplementary information Table S1 | Release specification of ^{89}ZED88082A

Test	Method	Specification
**Appearance**	Visual inspection	Colourless to light yellow
**pH**	Ph. Eur.	5.2 – 5.8
**Filter integrity**	Bubble point test	< 20%
**Strength**	Radioactive concentration	8.88 – 13.32 MBq/ml
**Purity**	SE-UPLC	RCP, main peak: ≥ 85.0%<br>RCP, radioactive HMWF: ≤ 10.0%<br>RCP, radioactive LMWF: ≤ 10.0%<br>Protein purity main peak: ≥ 95.0%
**Protein concentration**	UV-VIS	0.30 – 0.50 mg/ml
**Immunoreactive fraction**	SE-UPLC	≥ 70%
**Bacterial endotoxins**	Endosafe	< 2.5 EU/ml
**Sterility**[a]	Sterility test	No growth

[a]Post-release test; Results 14 days after inoculation.EU/ml: Endotoxin units per millilitre, HMWF: High molecular weight fraction, LMWF: Low molecular weight fraction, Ph. Eur.: European Pharmacopeia, RCP: Radiochemical purity, SE-UPLC: Size-exclusion ultra-performance liquid chromatography.
