# Supplementary material for: Whole-body CD8+ T cell visualization before and during cancer immunotherapy: a phase 1/2 trial
Source: Nat Med. 2022 Dec 5;28(12):2601–10. doi: 10.1038/s41591-022-02084-8 (PMC9800278; doi:10.1038/s41591-022-02084-8)
Supplement: Supplementary file 2 — Reporting Summary [file 41591_2022_2084_MOESM2_ESM.pdf]

nature portfolio

Corresponding author(s): E.G.E. de Vries

Last updated by author(s): 9/29/2022

Reporting Summary

Nature Portfolio wishes to improve the reproducibility of the work that we publish. This form provides structure for consistency and transparency in reporting. For further information on Nature Portfolio policies, see our Editorial Policies and the Editorial Policy Checklist.

Statistics

For all statistical analyses, confirm that the following items are present in the figure legend, table legend, main text, or Methods section.

n/a	Confirmed	
☐	☒	The exact sample size (n) for each experimental group/condition, given as a discrete number and unit of measurement
☐	☒	A statement on whether measurements were taken from distinct samples or whether the same sample was measured repeatedly
☐	☒	The statistical test(s) used AND whether they are one- or two-sided<br>*Only common tests should be described solely by name; describe more complex techniques in the Methods section.*
☐	☒	A description of all covariates tested
☐	☒	A description of any assumptions or corrections, such as tests of normality and adjustment for multiple comparisons
☐	☒	A full description of the statistical parameters including central tendency (e.g. means) or other basic estimates (e.g. regression coefficient) AND variation (e.g. standard deviation) or associated estimates of uncertainty (e.g. confidence intervals)
☐	☒	For null hypothesis testing, the test statistic (e.g. F, t, r) with confidence intervals, effect sizes, degrees of freedom and P value noted<br>*Give P values as exact values whenever suitable.*
☒	☐	For Bayesian analysis, information on the choice of priors and Markov chain Monte Carlo settings
☐	☒	For hierarchical and complex designs, identification of the appropriate level for tests and full reporting of outcomes
☐	☒	Estimates of effect sizes (e.g. Cohen's d, Pearson's r), indicating how they were calculated

Our web collection on statistics for biologists contains articles on many of the points above.

Software and code

Policy information about availability of computer code

Data collection	Siemens Biograph Vision PET/CT software versions CG70C/VG76A/VG80A, mCT PET/CT camera software versions VG70B/VG70C/VG60C, and Syngo.via version VB_40.02 (Imaging software). Image J version 1.52p (SDS-PAGE/autoradiography). Philips Intellisite Pathology solution v3.2 (IHC). OpenClinica version 3.14 (case record form)
Data analysis	R software version 4.1.1 for macOS, particularly using the lmer function for linear mixed models (lme4 1.1-27.1, lmerTest 3.1-3), coxphf for Cox models (coxphf 1.13.1), and rcspline.eval for restricted cubic splines (rms 6.2-0). Accurate tool for PET data analysis (versions .08072019, .22042020, and .14082020, RRID: SCR_020955). MATLAB version 2020b (Mathworks, Natick, MA, USA) for autoradiography and IHC analyses. Phoenix WinNonlin (Certara Inc., version 6.4) for pharmakinetic analyses. FlowJo v10 (Tree Star) for internalization analyses. Graphpad Prism v8.4.2.

For manuscripts utilizing custom algorithms or software that are central to the research but not yet described in published literature, software must be made available to editors and reviewers. We strongly encourage code deposition in a community repository (e.g. GitHub). See the Nature Portfolio guidelines for submitting code & software for further information.

Data

Policy information about availability of data

All manuscripts must include a data availability statement. This statement should provide the following information, where applicable:

- Accession codes, unique identifiers, or web links for publicly available datasets
- A description of any restrictions on data availability
- For clinical datasets or third party data, please ensure that the statement adheres to our policy

Clinical details of the cases and laboratory data, restricted to non-identifying data owing to privacy concerns, can be requested by e-mail from the corresponding author, who will handle all requests.

Field-specific reporting

Please select the one below that is the best fit for your research. If you are not sure, read the appropriate sections before making your selection.

☒ Life sciences ☐ Behavioural & social sciences ☐ Ecological, evolutionary & environmental sciences

For a reference copy of the document with all sections, see nature.com/documents/nr-reporting-summary-flat.pdf

Life sciences study design

All studies must disclose on these points even when the disclosure is negative.

Sample size	Exploratory first-in human, feasibility study with no pre-defined sample size, therefore no sample size calculations were performed.<br>In total 39 patients were included: 10 in part A, 29 in part B.<br>In part A, a minimum of two patients was required per dose cohort to determine the appropriate imaging dose and scanning time. Depending on the tumour saturation and scanning results, additional patients could be included. At least six patients would be enrolled at the dose level considered appropriate for further testing in part B.<br>In part B, inclusion of 30 patients was anticipated, with around 15-20 patients in part B1 who would be enrolled and treated with atezolizumab in the treatment trial, and approximately 10 patients with melanoma in part B2 who will be treated with standard of care PD-1 antibody therapy plus or minus ipilimumab. If a subject in cohort B was withdrawn from the study prior to accomplishment of the second ontreatment PET scan(s), an extra subject could be enrolled to generate sufficient data on ontreatment PET imaging, with a maximum total of 50 subjects for the whole study.
Data exclusions	One patient was excluded from PET and further analyses due to tracer extravasation.<br>Three patients were not evaluable for response: two because of early treatment termination for severe toxicity requiring high dose immunosuppressants, and one withdrew before starting treatment due to disease progression.<br>In seven out of 29 patients enrolled in part B, repeated PET imaging was not performed due to: withdrawal before (n = 1) and during treatment (n = 4) given disease progression, patient anxiety (n = 1), and COVID-19 restrictions (n = 1)<br>For tissue analyses, six out of 38 patients were excluded for baseline tumour tissue analyses due to: the planned biopsy not being performed for safety measures (n = 1), patient refused (n = 1), archival tissue was excluded due to intercurrent radiotherapy (n = 2), archival tissue was not retrievable anymore (n = 2). During treatment, 12 out of 29 patients were excluded for ontreatment tumour tissue analyses due to: biopsy not being performed because no participation in repeated PET procedures (n = 5), the biopsy not being performed for safety measures (n = 4), patient refused (n = 1), no suitable lesions due to intercurrent local radiotherapy (n = 2).
Replication	In cohort A, patients received 89ZED88082A/CED88004S once and underwent a PET scan on days 0, 2, 4 and 7. In cohort B, patients received 89ZED88082A/CED88004S twice, with a 30 days interval, followed by one to two PET scans on a different day. PET scans could not be repeated at the same time point at the same day in the same patient to avoid a too high patient burden given long scanning time.<br>All IHC was performed once per sample per staining, which was considered sufficient as IHC assays are validated assays which have been performed together with positive and negative controles. Autoradiography was performed only once per sample as patients were injected with 89ZED88082A and biopsied once, not allowing for replication due to decay of 89Zr. Radioactivity measurements and SDS-PAGE on blood samples were performed once per sample, as patients were injected with 89ZED88082A once and blood was collected once per time point and used for several analysis. 89ZED88082A internalization in healthy donor PBMCs was assessed once in 2 technical replicates. Analyses on presences of ADAs was assessed once per sample. ADAs were examined before tracer injection (n = 31), 28-50 days after the first injection (n = 26) and 18-38 days after the second injection (n = 12). TBNK enumeration was performed once per sample per timepoint.
Randomization	Randomization was not performed in this trial as it was a first-in-human feasibility trial and all patients received treatment with immune checkpoint inhibitors after molecular PET imaging. Cohort allocation was performed based on order of enrollment.
Blinding	Blinding for treatment was not necessary in this trial, as all patients received treatment with immune checkpoints inhibitors after molecular PET imaging in the companion treatment study, or as standard of care if applicable. CD8 expression profile based on IHC was performed by a pathologist blinded for treatment outcome. Three of six investigators doing PET quantification were not blinded to dose cohort allocation or clinical outcomes, as they were simultaneously clinically involved in patient care. However supervisors of quantification results were blinded. The study personal who performed the laboratory assessments were blinded for dose cohort allocation and clinical outcomes.

Reporting for specific materials, systems and methods

We require information from authors about some types of materials, experimental systems and methods used in many studies. Here, indicate whether each material, system or method listed is relevant to your study. If you are not sure if a list item applies to your research, read the appropriate section before selecting a response.

Materials & experimental systems

n/a	Involved in the study
☐	☒ Antibodies
☒	☐ Eukaryotic cell lines
☒	☐ Palaeontology and archaeology
☒	☐ Animals and other organisms
☐	☒ Human research participants
☐	☒ Clinical data
☒	☐ Dual use research of concern

Methods

n/a	Involved in the study
☒	☐ ChIP-seq
☐	☒ Flow cytometry
☒	☐ MRI-based neuroimaging

Antibodies

Antibodies used

Study drug; CED88004S (Genentech developed and owns the intellectual property rights pertaining to CED88004S).
Mouse anti-CD8 monoclonal antibody clone C8/144B (DAKO/Agilent; M7103) was used for IHC. For immune cell characterization using flow cytometry, peridinin chlorophyll protein complex-cyanine5.5 (PerCP/Cy5.5)-conjugated mouse anti-human CD3 monoclonal antibody clone OKT3 (Thermofisher Scientific; 45-0037-42) and allophycocyanin (APC)-conjugated donkey anti-human IgG F(ab')2 fragment (Jackson ImmunoResearch Laboratories; 709-136-149) were used.

Validation

CED88004S: An enzyme linked immunosorbent assay (ELISA) was used to examine the binding of CED88004S to recombinant human CD8 Fc fusion protein. ELISA results demonstrated that CED88004S (DFAR ranging from 1.0-1.5) binds with mean (±SD) EC50 (ng/mL) of 47.7 (±5.5) - 49.1 (±2.1) to CD8. SD = standard deviation; DFAR = DFO to antibody ratio, 1.0-1.5 is within acceptance criteria. For extended non-clinical pharmacology related to CED88004S, please refer to Gill et al., AAPS J 2020 (PMID: 31900688).
Anti-CD8 mAb DAKO/Agilent; Optimized for IHC with validated protocols (Ref. statement on website: https://www.agilent.com/en/product/immunohistochemistry/antibodies-controls/primary-antibodies/cd8-(concentrate)-76631#productdetails).
Anti-CD3 mAb Thermofisher Scientific; The OKT3 clone is commonly reported for use in flow cytometric analysis (HCDM database: https://hcdm.org/index.php/molecule-information?view=molecule&task=viewmolecule&moleculeid=227&search=cd3).
Anti-IgG F(ab')2 fragment Jackson ImmunoResearch Laboratories; According to product specifications, the antibody reacts with whole molecule human IgG based on immunoelectrophoresis and/or ELISA. It also reacts with the light chains of other human immunoglobulins. No antibody was detected against non-immunoglobulin serum proteins. The antibody has been tested by ELISA and/or solid-phase adsorbed to ensure minimal cross-reaction with bovine, chicken, goat, guinea pig, Syrian hamster, horse, mouse, rabbit, rat, and sheep serum proteins, but it may cross-react with immunoglobulins from other species.

Human research participants

Policy information about studies involving human research participants

Population characteristics

Eligible patients for part A or B1 had a histologically confirmed locally advanced or metastatic cancer, whom, in the investigator's opinion, based on available clinical data, may benefit from anti-PD-L1 antibody treatment. They were required to have disease progression during or following first-line standard-of-care therapy. In part B2, patients with melanoma eligible for standard-of-care anti-PD-1 antibody with or without ipilimumab, could participate. Eligible patients had measurable disease according to RECIST1.1, and were amenable to a tumour biopsy. All patients were ≥ 18 years of age and had an Eastern Cooperative Oncology Group performance status of 0–1, life expectancy ≥ 12 weeks, and adequate hematologic and end-organ function. Patients with concomitant or historical conditions or medication use that could compromise their safety with 89ZED88082A/CED88004S or atezolizumab treatment, or interpretation of study results, were excluded. Characteristics of evaluable patients (n = 38): median age 62 years (ranging 32-80), 53% female (n = 20). Tumour types included: 9 mismatch repair protein deficient (5 colorectal, 2 urothelialcell, 1 duodenal, 1 pancreatic), 5 cervical carcinoma, 4 cutaneous squamous cell carcinoma, 3 triple negative breast cancer, 3 cholangiocarcinoma, 3 melanoma, 2 anorectal squamous cell carcinoma, 2 vulvar squamous cell carcinoma, 2 neuroendocrine carcinoma (cervical, gastric-oesophageal), 1 oesophageal squamous cell carcinoma, 1 non-small cell lung cancer, 1 hepatocellular carcinoma, 1 ovarian clear cell carcinoma, 1 squamous cell carcinoma of unknown primary.

Recruitment

Potential participants were suggested to the study team by the threating physician. Before agreement to participation in this trial, all patients had been provided with written information in the form of a Patient Information Sheet, which was approved by the Medical Ethical Committe. Potential participants were orally informed about the study (including the aim of the study, possible AEs and the procedure) and asked for their consent by a medical doctor after max. 1 week of consideration. Each patient was given the opportunity to ask questions and was informed about the right to withdraw from the study at any time. Healthy blood donors were xxxx

Ethics oversight

Medical Ethical Committee of the University Medical Center Groningen. Central Committee on Research Involving Human Subjects of the Netherlands.

Note that full information on the approval of the study protocol must also be provided in the manuscript.

Clinical data

Policy information about clinical studies
All manuscripts should comply with the ICMJE guidelines for publication of clinical research and a completed CONSORT checklist must be included with all submissions.

Clinical trial registration	NCT04029181. Companion atezolizumab treatment study: NCT02478099.
Study protocol	The complete study protocol can be requested by e-mail from the corresponding author, who will handle all requests.
Data collection	The study was performed at the University Medical Center Groningen, the Netherlands. Patients were enrolled between February 2019 and November 2020.
Outcomes	1. Primary outcomes. [a]. Safety assessment through summaries of adverse events, changes in laboratory test results (if evaluation is indicated), changes in vital signs, and exposure to ZED88082A/CED88004S. Adverse event data will be recorded and summarized according to NCI CTCAE v4.0. [b]. Appropriate dosing and imaging time points of the anti-CD8 imaging agent will be determined based on measurements of standardised uptake value (SUV) of defined volumes of interest (VOIs) on the immunoPET scan images. [c]. Description of PK of the anti-CD8 imaging agent by measuring standardised uptake value (SUV) on PET scans performed 0, 2, 4 and/or 7 days after tracer injection before and during atezolizumab or PD-1 antibody immune checkpoint inhibitor plus or minus ipilimumab treatment. [d]. Assessment of the immunogenic potential of the anti-CD8 imaging agent by measuring incidence of anti-drug antibodies during the study relative to the prevalence of ADAs at baseline and assessing their relationship to other outcomes measured.<br>2. Seconday outcomes: [a]. Heterogeneity of imaging tracer uptake will be evaluated by measuring standardised uptake value (SUV) in defined volumes of interest (VOIs) of tumour lesions on the immunoPETscan images. [b]. Normal organ uptake of the anti-CD8 imaging agent as measured by SUVs on PET scan images will be analyzed on correlation to (serious) adverse events (possibly) related to ICI treatment, defined as all (S)AEs which are assessed as "possibly", "probably" or "definitely" related to ICI treatment. [c]. Results of immunohistochemical scoring of tumour and immune cell CD8 and other markers of lymphocytic infiltration in fresh biopsies will be described as a semi-quantitative score using the percentage of positive cells (continuous variable), intensity and pattern of staining (discrete variable). These IHC results will be compared with imaging tracer standardised uptake value (SUV) in defined volumes of interest (VOIs) of tumour lesions on the immunoPETscan images. Results of autoradiography will be described by measuring standardised uptake value (SUV) on the biopsy slides.

Flow Cytometry

Plots

Confirm that:

☒ The axis labels state the marker and fluorochrome used (e.g. CD4-FITC).

☒ The axis scales are clearly visible. Include numbers along axes only for bottom left plot of group (a 'group' is an analysis of identical markers).

☒ All plots are contour plots with outliers or pseudocolor plots.

☒ A numerical value for number of cells or percentage (with statistics) is provided.

Methodology

Sample preparation	PBMCs were prepared from healthy blood donor buffy coats (Sanquin) with appropriate informed consent by Ficoll gradient centrifugation in Leucosep tubes (Greiner Bio-One).
Instrument	Samples were analyzed on a BD FACS Verse flow cytometer (BD Biosciences).
Software	Data analysis was performed with FlowJo v10 (Tree Star).
Cell population abundance	At least 10,000 events were measured within the CD3+ cell population. CED88004S membrane binding is expressed as mean fluorescent intensity (MFI), and no cell sorting was applied.
Gating strategy	PBMCs were gated in a forward scatter (FSC) versus side scatter (SSC) dot plot. Lymphocytes were gated in an FSC versus SSC dot plot, doublets were excluded by plotting FSC height (FSC-H) versus area (FSC-A). CD3 positive T cells were gated on the anti-human peridinin chlorophyll protein complex-cyanine5.5 (PerCP/Cy5.5)-CD3 staining. Binding/internalization of CED88004S was detected using an anti-human allophycocyanin-IgG F(ab')2 fragment within the total PBMC population or CD3-positive cell population. Samples were measured in duplicate and corrected for background fluorescence and non-specific antibody binding.

☒ Tick this box to confirm that a figure exemplifying the gating strategy is provided in the Supplementary Information.
