# Supplementary figures and images for: Whole-body CD8+ T cell visualization before and during cancer immunotherapy: a phase 1/2 trial
Source: Nat Med. 2022 Dec 5;28(12):2601–10. doi: 10.1038/s41591-022-02084-8 (PMC9800278; doi:10.1038/s41591-022-02084-8)

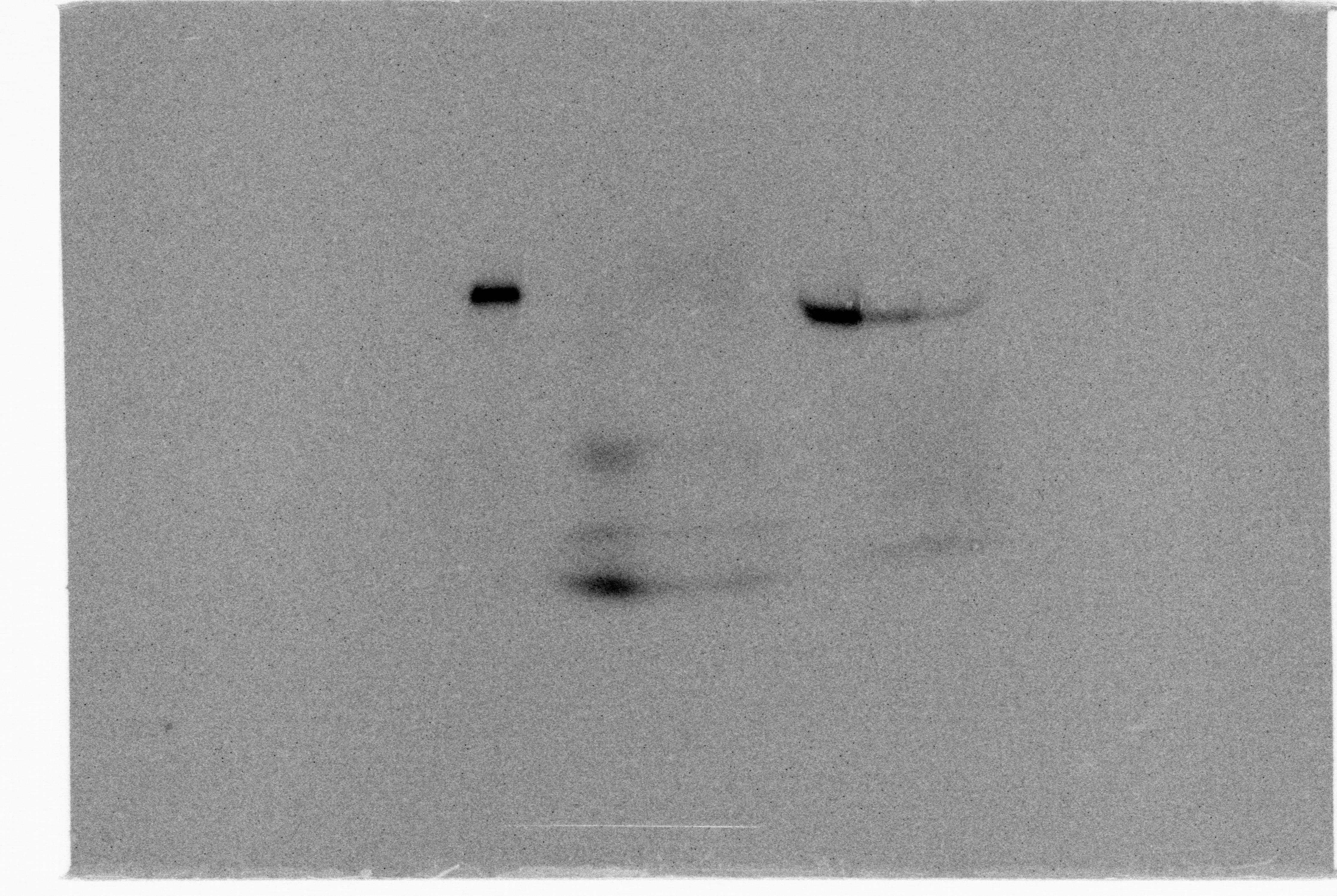

Supplement: Supplementary file 4 — Unprocessed SDS–PAGE combined with autoradiography. [file 41591_2022_2084_MOESM4_ESM.tif]

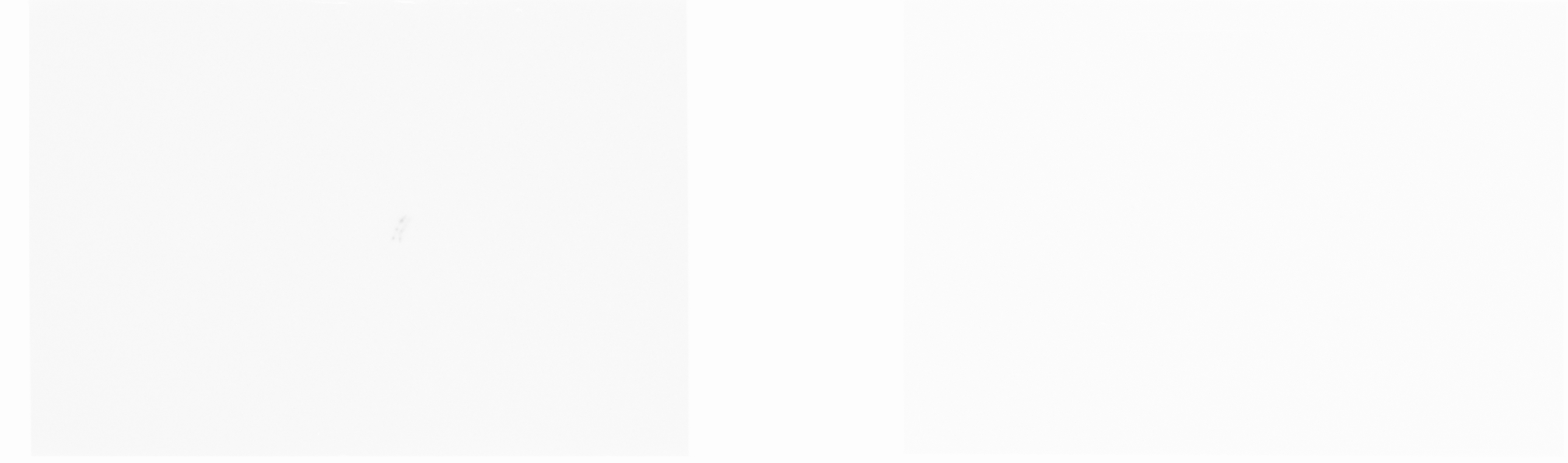

Supplement: Supplementary file 5 — Unprocessed tumor autoradiography image. [file 41591_2022_2084_MOESM5_ESM.tif]
